# Supplementary material for: Rapid handheld time-resolved circularly polarised luminescence photography camera for life and material sciences
Source: Nat Commun. 2023 Mar 20;14:1537. doi: 10.1038/s41467-023-37329-8 (PMC10027819; doi:10.1038/s41467-023-37329-8)
Supplement: Supplementary file 1 — Supplementary Information file [file 41467_2023_37329_MOESM1_ESM.pdf]

Supplementary Information

Rapid handheld time-resolved circularly polarised luminescence photography camera for life and material sciences

Davide F. De Rosa,[1] Patrycja Stachelek,[1] Dominic J. Black,[1] Robert Pal[1,*]

[1] Department of Chemistry, Durham University, South Road, Durham, United Kingdom, DH1 3LE.

*Email of corresponding author: robert.pal@durham.ac.uk

Table of Contents

Experimental Information 2

Synthetic Procedures 9

Thin film preparation 9

Supplementary Figures 10

Absorption, Emission, Excitation, and CPL Spectra 10

References 52

Synthesis of pyridyl arm precursor 26

Synthesis of extended chromophore precursor 28

Synthesis of [Eu:L1] 30

NMR Spectra 36

Experimental Information

Absorption and emission spectroscopy

Absorption spectra were recorded using ATI Unicam UV/Vis spectrometer (Model UV2) using Vision 3.33 software. All the steady-state emission and excitation spectra were recorded using ISA Jobin-Yvon Spex Fluorolog-3 luminescence spectrometer using DataMax v2.2 software. Samples were held within 1 cm path length quartz cuvettes.

Lifetime measurements were carried out with a Perkin Elmer LS55 spectrometer using FL Winlab software. Quantum yield measurements were calculated by comparison with two standards rhodamine B ($C_{28}H_{31}ClN_2O_3$) and fluorescein ($C_{20}H_{12}O_5$). For the standards and each of the unknowns, five solutions with absorbance values between 0.05 and 0.1 were used. The quantum yield was calculated according to the equation:

$$\Phi_x = \Phi_r \cdot \frac{A_r}{A_x} \cdot \frac{E_x}{E_r} \cdot \frac{I_r}{I_x} \cdot \frac{\eta_x^2}{\eta_r^2} \qquad \text{(Supplementary Eq. 1)}$$

where *r* and *x* refer to reference and unknown respectively; *A* is the absorbance at λ_{ex} *; E* is the corrected integrated emission intensity*; I* is the corrected intensity of excitation light*; h* is the refractive index of solution.

CPL spectroscopy

PEM-CPL spectrometer:[3] CPL was measured with a home-built (modular) spectrometer. The excitation source was a broad band (200 – 1000 nm) laser- driven light source EQ 99 (Elliot Scientific). The excitation wavelength was selected by feeding the broadband light into an Acton SP-2155 monochromator (Princeton Instruments); the collimated light was focused into the sample cell (1 cm quartz cuvette). Sample photoluminescence (PL) emission was collected 90° to the excitation direction with a lens (f = 150 mm). The emission was fed through a photoelastic modulator (PEM) (Hinds Series II/FS42AA) and through a linear sheet polariser (Comar). The light was then focused into a second scanning monochromator (Acton SP-2155) and subsequently onto a photomultiplier tube (PMT) (Hamamatsu H10723 series). The detection of the CPL signal was achieved using the field modulation lock-in technique. The electronic signal from the PMT was fed into a lock-in amplifier (Hinds Instruments Signaloc Model 2100). The reference signal for the lock-in detection was provided by the PEM control unit. The monochromators, PEM control unit, and lock-in amplifier were interfaced to a desktop PC and controlled by a custom-written LabVIEW graphic user interface. The lock-in amplifier provided two signals, an AC signal

corresponding to (I_L- I_R) and a DC signal corresponding to (I_L + I_R) after background subtraction. The emission dissymmetry factor was therefore readily obtained from the experimental data, as 2 AC/DC.

Spectral calibration of the scanning monochromator was performed using a Hg-Ar calibration lamp (Ocean Optics). A correction factor for the wavelength dependence of the detection system was constructed using a calibrated lamp (Ocean Optics). The measured raw data was subsequently corrected using this correction factor. The validation of the CPL detection systems was achieved using light emitting diodes (LEDs) at various emission wavelengths. The LED was mounted in the sample holder and the light from the LED was fed through a broad band polarising filter and $\lambda/4$ plate (Ocean Optics) to generate circularly polarised light. Prior to all measurements, the $\lambda/4$ plate, and an LED were used to set the phase of the lock-in amplifier correctly. The emission spectra were recorded with 0.5 nm step size and the slits of the detection monochromator were set to a slit width corresponding to a spectral resolution of 0.25 nm. CPL spectra (as well as total emission spectra) were obtained through an averaging procedure of several scans. The CPL spectra were smoothed using a shape-preserving Savitzky-Golay smoothing (polynomial order 5, window size 9 with reflection at the boundaries) to reduce the influence of noise and enhance visual appearance; all calculations were carried out using raw spectral data. Analysis of smoothed vs raw data was used to help to estimate the uncertainty in the stated g_{em} factors, which was typically ±10%.

SS-CPL spectrometer:[4] Sample excitation was provided by a 2.35 W 365 nm LED with a 9 nm spectral half-width [CUN6AF4A; Roithner LaserTechnik] mounted in a custom-built heat sink. The LED was powered using a bench-top power supply [PL303QMD 30V/3A; Aim TTI] operating in constant current mode. A collimating lens, ground-glass diffuser [N-BK7, Thorlabs], and a 240 – 395 nm bandpass filter [FGUV5, Thorlabs] placed prior to the sample ensured that excitation light was diffuse, unpolarised, and constrained to $\lambda < 400$ nm. Samples were placed within an enclosed holder [CVH100, Thorlabs]. All samples were measured in quartz cuvettes with a 10 mm by 10 mm cross-section [111-10-40; Hellma Analytics]. Sample emission was collected 90° to the excitation beam. The emitted light passed through an achromatic QWP [AQWP05M-600; Thorlabs] in order to convert circularly polarised light into two orthogonal, linearly polarised, signals, corresponding to L-CPL and R-CPL. The light then was then split into two spatially separated detection channels by a non-polarising 50/50 beam splitter [BS013; Thorlabs]. Each detection channel was capable of independently measuring L-CPL and R-CPL in a sequential manner by precisely rotating a linear polariser [LPVISE100-A; Thorlabs] mounted in a motorized precision rotation mount [PRM1/MZ8; Thorlabs] controlled via an electronic controller [KDC101; Thorlabs]. A long pass filter ($\lambda > 450$ nm) [FEL0450, Thorlabs] prior to detection ensured no stray excitation light could reach the detector. Emission intensities of left CPL and right CPL channels were quantified by two matched high-sensitivity solid-state (SS) charge coupled device (CCD) spectrometer operating at 400 – 800 nm with ~0.2

nm sampling increments [Ocean Optics Maya2000Pro, H3 grating, 350 – 850 nm]. To maximise light throughput to the CCD detector, the spectrometer entrance aperture slit was removed.

From the intensity of each channel, the total emission $(I_{L-CPL} + I_{R-CPL})$, CPL $(I_{L-CPL} - I_{R-CPL})$ spectra and g_{em} (emission dissymmetry factor) was calculated as,

$$g_{em} = \frac{2(I_{L-CPL} - I_{R-CPL})}{(I_{L-CPL} + I_{R-CPL})} \qquad \text{(Supplementary Eq. 2)}$$

Synchronised operation of both channels enabled rapid concurrent acquisition of full CPL spectra. SS-CPL operation was automated using custom-written LabVIEW programs [LabVIEW 2013, National Instruments].[5] Data was analysed post-hoc with custom-written Matlab programs [Matlab 2019a, MathWorks].

Time-resolved detection of whole-spectra was achieved by introducing a time delay between the pulsed LED excitation and the start of SS-CCD spectrometer acquisition. LED pulsation and spectrometer acquisition were synchronised to transistor–transistor logic (TTL) signals produced via a USB multifunction I/O device [USB-6210, National Instruments]. Acquisition at a rate of 43 Hz was found to minimize spurious noise (*e.g.* from mains frequency electronics at 50 Hz) whilst providing a suitable measurement window for time-resolved measurement of long-lived lanthanide emission (*i.e.* several milliseconds) with a 10-millisecond integration time.

MP-Spectroscopy: Two photon CPL spectroscopy has been achieved by coupling (beam routing using mirrors, Thor Labs BB1-E03) a tuneable femtosecond pulsed laser (680 – 1300 nm, Coherent Discovery TPC, 100 fs, 80 MHz) to two pre-existing CPL spectrometers detailed above. Initial proof of concept two photon spectroscopy has been achieved by perpendicularly mounting an Ocean Optics HR2000Pro (2048-pixel linear CCD Sony ILX5 chip, 200 µm slit, H3 grating, 350 – 850 nm spectral region) spectrometer as a 'third arm' to the Discovery TPC laser. The laser beam was focused onto the centre of the 1 cm path sample holder (Thor labs CVH100) by a dedicated ultrafast laser lens (Edmund Optics 11711, 50 mm focal length). The spectrometer has also been equipped with a perpendicularly mounted 365 nm LED (nichia, 1W) and been operated using a modified version of the above-mentioned custom time resolved detection and accumulation algorithm written in Labview2013 program.

In order to eliminate unwanted artefacts associated with stray light from MP excitation each spectrometer have been equipped with a rotating filter wheel (Thor Labs, CFW6) housing an LP420 (Comar Optics, for 365 nm UVLED excitation) and SP650 and SP700 (Edmund Optics, 8472 and 8474 for MP excitation) filters.

Enantioselective CPL-LSCM microscopy imaging:[6] CPL-LCSM was enabled by adapting a commercial laser scanning confocal microscope (LSCM) (SP5 II, Leica Microsystems) with excitation provided by a fibre coupled 80 mW variable power 355 nm Nd:YAG CW laser. The CPL analysis module was external to an output port and all elements were mounted in a 30 mm cage mount system for optimal alignment (assorted 30 mm components, Thorlabs). First, light from the sample plane of the LSCM exits the microscope via an output port and into an external CPL analysis module, which is adapted from the previously described rapid SS-CPL spectrometer.[4,6] First, the waveband of interest is selected by a switchable bandpass filter mounted into a motorised 1" filter wheel (FW102C, ThorLabs). The two main filters used for Eu(III) emission, tailored to ΔJ = 1 and 2 bands, are BP594/10 and BP610/10 (86737 and 65164, Edmund optics, O.D. 4.0) respectively. Chromatically filtered light then passes through an achromatic quarter wave plate (AQWP05M-600, Thorlabs) where it converts left and right circularly polarised light into orthogonal linearly polarised states. The light is then split into two analysis pathways by a non-polarising 50:50 beam-splitter cube (BS013, Thorlabs). The two linear polarised light states generated (horizontal and vertical polarisation) corresponding to left or right CPL are selected by a carefully aligned linear polarizer, housed in a high-precision computer-controlled rotation mount (LPVISE100-A, Thorlabs) orientated to select for left or right CPL states via computer control software (Kinesis, Thorlabs). The emission intensity of each pixel is quantified in a conventional LSCM scanned manner by a dedicated high sensitivity avalanche photodiode pair (Leica ADPs, Becker & Hickl ID-120). Whilst each detection arm can operate independently, both components are matched in alignment and specification to enable rapid and simultaneous acquisition of left and right CPL images. The two detection arms were aligned to achieve matched sensitivity to enable rapid simultaneous acquisition of left and right CPL images. Calibration of the linear polarisers for enantioselective localisation was executed based upon the procedure reported in Mackenzie et al.[4]

EDCC imaging was realised by subtraction of the simultaneously recorded left-handed CPL image from the right-handed CPL (and vice versa) using ImageJ software (v1.49).

Enantioselective epifluorescence microscopy imaging:[1] Chiroptical contrast-based imaging, *i.e.* the separation of left- and right-handed circularly polarised light emitted by the separate Λ- and Δ-enantiomers of a suitable Eu:L4 has been facilitated via modification of a time-resolved Zeiss Axiovert 200M epifluorescence microscope set-up.[5] Areas of the two enantiopure spots with equal brightness were selected (contrast values: Λ-[Eu:L4] 222/255 and Δ-[Eu:L4] 231/255) and using a dye punch (0.3 x 150 mm), two well-defined line-shaped Eu:L4 blotted pieces of paper were mounted parallel to each other onto a SuperFrost microscope slide capped with a 170 µm coverslip for imaging. The microscope is equipped with a variable pulse sequence generator, which allows both CW and time-resolved operation. Acquisition using an EOM (monochrome, ThorLabs) 0.7 megapixel rolling shutter camera was set at 7.2

ms per frame, and a typical value for time gating was 20 µs, after pulsed excitation with a 365 nm UV LED (24 V, 1.2 W, collimated and scrambled to 1" diameter, focused to the back focal plane (BFP) of Zeiss x10/0.25NA A-Plan air objective). The sequence can be programmed for any number of frame averages or, for low light collection, accumulation, controlled manually or by an average FOV pixel contrast saturation-limiting algorithm. This camera has been successfully substituted with the CPLP camera reported herein (see main text *Methods* section for full description).

CPLP: An important consideration when custom triggering of the camera is required, is to interface the camera with the desired control and automation software (*i.e.* LabView) and/or signal generator module by keeping a few important hardware driven facts under control. External triggering enables the camera to be easily integrated into systems that require the camera to be synchronized to external signal generators or strobe set up (pulsed excitation), to facilitate accumulated unaveraged image acquisition and signal build up. We would guide the reader to familiarise themselves with the camera (Kiralux™) manufacturers website (https://www.thorlabs.com/newgrouppage9.cfm?objectgroup_id=13033) in order to gain full appreciation on the possibilities and pitfalls of external triggering and time-resolved operation. The following important aspects needs to be considered. Due to the general operation of CMOS sensor cameras, as well as typical system propagation delays, the actual timing relationships of the camera are subject to the following considerations:

The delay from the external trigger to the start of the exposure and strobe signals is typically 100 ns for all triggered modes (standard and PDX/Bulb).

For PDX/Bulb mode (later used herein) triggered exposures, in addition to the 100 ns delay at the start of the exposure, there is also a 13.72 µs integration period AFTER the falling edge of the external trigger. This is inherent in the sensor operation. It is important to note that the Strobe_out signal includes the additional 13.72 µs integration time and therefore is a better representation of the actual exposure time. Our suggestion is to use the Strobe_out signal to measure your exposure time and adjust your PDX mode trigger pulse accordingly.

Cross section determination

The cross-section (σ^2) of the standard materials discussed herein,[1,7] is calculated according to established procedures:[8]

$$\frac{\sigma_2^S \phi^S}{\sigma_2^S \phi^S} = \frac{C_R n_S F^S(\lambda)}{C_S n_R F^R(\lambda)}. \qquad \text{(Supplementary Eq. 3)}$$

Where *S* is sample, *R* is reference, ϕ is the total emission quantum yield of the compound (composite photoluminescence of both chromophore and Eu(III) ion for ϕ_S), *C* is the concentration, *n* the refractive

index and $F^S(\lambda)$ and $F^R(\lambda)$ are the integrated PL spectrum for the sample and reference, respectively. Additionally, the two-photon nature of the excitation process was proven by recording an excitation power dependence; the resulting line has a slope of two on a logarithmic scale.[9] The cross sections were calculated with reference to Rhodamine B in ethanol. The MP-CPL spectrum was recorded using 6 µM solutions in 1 cm path length quartz cuvettes.

General Synthetic Procedures

Commercially available reagents were used as received. Solvents were laboratory grade and were dried over appropriate drying agents when required. Where appropriate, solvents were degassed using freeze-pump-thaw cycles.

Thin layer chromatography (TLC) was carried out on aluminium-backed silica gel plates with 0.2 mm thick silica gel 60 F254 (Merck) and visualised by UV irradiation at 254 nm or 366 nm. Preparative flash column chromatography was performed using flash silica gel 60 (230-400 mesh) from Merck or Fluorochem.

^{1}H, ^{13}C, and ^{31}P NMR spectra were recorded in commercially available deuterated solvents on a Bruker Avance-400 (^{1}H at 400.06 MHz, ^{13}C at 100.61 MHz, and ^{31}P at 161.95 MHz), a Mercury 400 (^{1}H at 399.95 MHz), a Varian VNMRS-600 (^{1}H at 599.67 MHz, ^{13}C at 150.79 MHz and ^{31}P at 242.75 MHz), or a Varian VNMRS-700 (^{1}H at 699.73 MHz, ^{13}C at 175.95 MHz and ^{31}P at 283.26 MHz). All chemical shifts are in ppm and coupling constants are in Hz.

Electrospray mass spectra were obtained on a TQD mass spectrometer equipped with an Acquity UPLC system, an electrospray ion source, and an Acquity photodiode array detector (Waters Ltd., UK). Accurate masses were recorded on an LCT Premier XE mass spectrometer or a QToF Premier Mass spectrometer, both equipped with an Acquity UPLC, a lock-mass electrospray ion source and an Acquity photodiode array detector (Waters Ltd., UK). MeOH or MeCN were used as the carrier solvents.

HPLC Analysis

Reverse-phase HPLC was performed at 295 K using a Shimadzu system comprising of a Degassing Unit (DGU-20A5R), a Prominence Preparative Liquid Chromatography pump (LC-20AP), a Prominence UV-Vis Detector (SPD-20A), and a Communications Bus Module (CBM-20A). A preparative XBridge C18 OBD column was used (19 × 100 mm, 5 µm) with a flow rate of 17 mL min^{-1}. Fraction collection was performed manually. The water / MeCN solvent system used to achieve purification is detailed in Supplementary Table 1.

Step	Time / min	% H2O	% MeCN
0	0	90	10
1	4	90	10
2	14	0	100

3	19	0	100
4	22	90	10

Supplementary Table 1. Details of the water / MeCN solvent system used in gradient elution to achieve purification.

Chiral HPLC analysis was carried out on a Perkin Elmer Series 200 system comprising of a Perkin Elmer Series 200 pump, autosampler, and UV-Vis detector, using a Daicel CHIRALPAK-IC or ID column (4.6 × 250 mm for analytical with a flow rate of 1.0 mL min-1, 10 × 250 mm for preparative with a flow rate of 4.4 mL min-1, all 5 µm particle size). Isocratic methanol was used as the mobile phase. Fraction collection was automated.

Chiral HPLC analysis and purification were carried out on a Perkin Elmer Series 200 system comprising of a Perkin Elmer Series 200 pump, autosampler, and UV-Vis detector, using a Daicel CHIRALPAK-IC column (4.6 × 250 mm for analytical with a flow rate of 1.0 mL min-1, 10 × 250 mm for preparative with a flow rate of 4.4 mL min-1, all 5 µm particle size). The chiral stationary phase of the Daicel CHIRALPAK-IC column, *i.e.* cellulose tris(3,5-dichlorophenylcarbamate, is shown in Supplementary Figure 1. Isocratic methanol was used as the mobile phase. Fraction collection was automated.

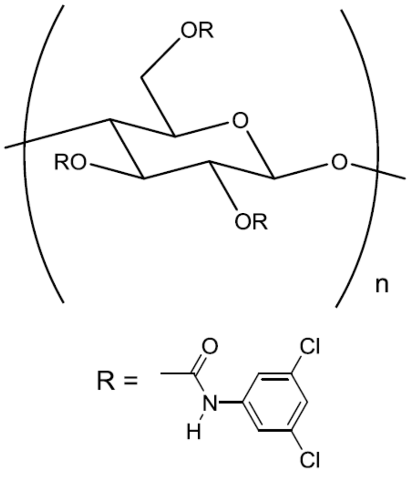

Cellulose tris(3,5-dichlorophenylcarbamate)

Supplementary Figure 1. The chiral stationary phases used in Daicel CHIRALPAK-IC column.

Synthetic Procedures

Film preparation

Drop casting procedure

Films were prepared by drop casting lanthanide complexes in poly(methylmethacrylate) (PMMA) (M_w ~ 15000 g/mol, Sigma-Aldrich) matrix as follows. The polymer was stirred in dichloromethane (DCM) (40 mg/ml) and refluxed at 40°C for 15 minutes. The lanthanide complex was dissolved in DCM and added to the polymer solution and refluxed at 40°C for 5 minutes. A layer of the solution of the above mixture was deposited on a glass substrate using a glass Pasteur pipette. The carrier solvent was allowed to evaporate at room temperature to afford a film.

Spin coating procedure

Films were prepared by spin coating lanthanide complexes in poly(vinylpyrrolidone) (PVP) (M_w ~ 40000 $gmol^{-1}$, Sigma-Aldrich) matrix as follows. The polymer was dissolved in methanol (MeOH) (55 mg/ml) and left stirring overnight at room temperature. The lanthanide complex was dissolved in MeOH. 500 µl of the PVP solution was subsequently mixed with 100 µl of the lanthanide complex solution. Three layers of the solution of the above mixture were deposited on a glass substrate at 1,500 RPM for 1 minute. The resulting film was annealed on a hot plate for 5 – 10 minutes at 35°C after each layer was deposited to ensure the absence of cracks on the surface of the film.

The thickness of drop cast and spin coated film was verified using PhMoNa enhanced Laser Scanning Confocal Microscopy capable of achieving 380 nm axial resolution using a x63 NA objective and a 355 nm (Nd:YAG laser, 3rd harmonic) excitation wavelength.[10] Thickness determination of the films was performed via fluorescence emission tracking using diffraction-limited z-stack images. Films thickness was maintained around 15 ± 3 micron (µm) using set viscosity controlled through the carrier solvent. Importantly, these imaging experiments also confirmed the desired homogeneous distribution of the fluorophore embedded in the polymer matrix. Inhomogeneity in the edge of the glass slides caused internal reflections that resulted in phase-inversion and degradation of CPL signal. For this reason, only the bulk of these test-targets was considered suitable for the purposes of demonstrating CPL-LSCM. The edge of the samples was discarded for analysis and, accordingly, the samples could not be used for side-by-side comparisons.

Supplementary Figures

Absorption, Emission, Excitation, CPL spectra, 2PE studies, EDCC images and CPLP imaging sequences

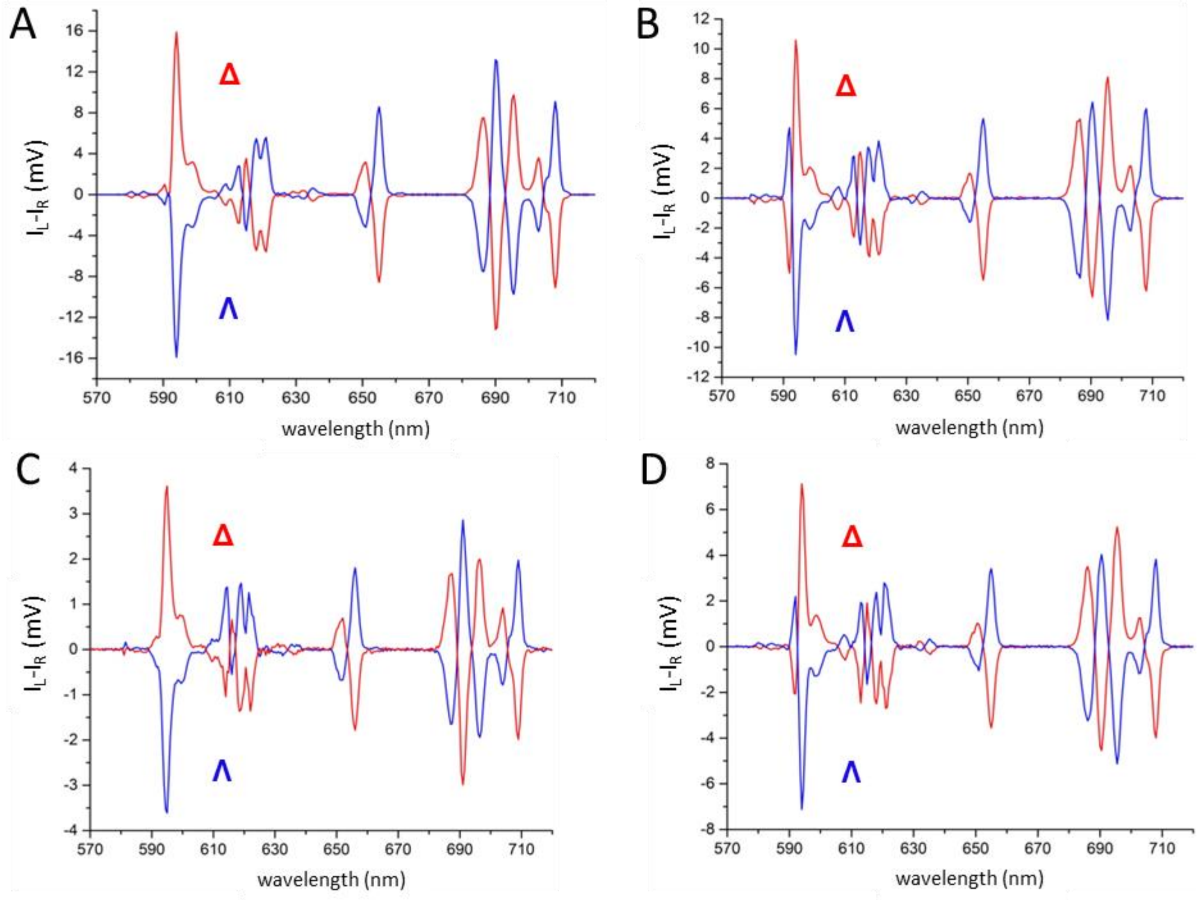

ΔJ=x

Supplementary Figure 2. CPL spectra of the heritage samples used for TR-CPLP. CPL emission spectra of Δ- (red) and Λ- (blue) enantiomers of (A) Eu:L2, (B) Eu:L3, (C) Eu:L4, (D) Eu:L5. All spectra were recorded in MeOH, 295 K, 5 μM complex, λ_{ex} = 365 nm, 5 scans averaged with 0.5 nm resolution and 1 ms integration time (C = 5 × 10^{-5} M).

Complex	ε / cm^{-1}M^{-1}	Total Φ_{em}	ΔJ=x Φ_{em}	$\lvert g_{em}(I_{max}, \Delta J{=}x) \rvert$	$CPB^{\Delta J=x}_{365\,nm}$ / cm^{-1}M^{-1}	τ / ms
**Eu:L1**	40000	0.47	**ΔJ=1** 0.043	0.20 (0.28)*	172 (241)*	0.97
**Eu:L1**	40000	0.47	**ΔJ=2** 0.132	0.03 (0.05)*	79 (132)*	0.97
**Eu:L2**	60000	0.50	**ΔJ=1** 0.045	0.13	176	1.22
**Eu:L3**	60000	0.42	**ΔJ=1** 0.038	0.12 (0.21)*	137 (240)*	1.14
**Eu:L3**	60000	0.42	**ΔJ=2** 0.118	(0.02)*	(71)*	1.14
**Eu:L4**	60000	0.39	**ΔJ=1** 0.035	0.14	147	1.18
**Eu:L5**	60000	0.28	**ΔJ=1** 0.025	0.14	105	1.10
**Tb:L6**	8300	0.40	**ΔJ=5** 0.144 (0.075)#	0.27	161 (84)#	1.56

Supplementary Table 2. Key photophysical parameters for the Eu:L1-5 and Tb:L6 in MeOH and (PMMA)*****, λ_{exc} = 365 nm. #Corresponding Tb:L6 values in bracket are the calculated true values taking intra-transitional CPL sign cancellation into account. For relevant chemical structures please see main Figure 1 and Supplementary Figure 21.

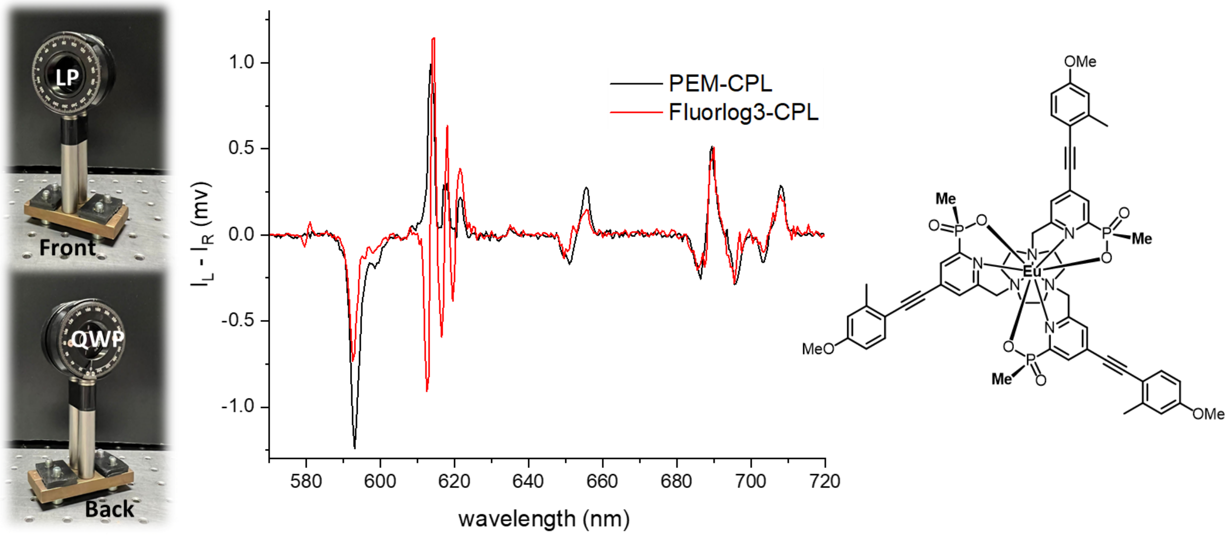

Supplementary Figure 3. Comparison of CPL spectra recorded using an adapted commercial fluorimeter and a custom-built CPL spectrometer. (Red) CPL emission spectra of Λ-Eu:L3 (C = 5 × 10^{-5} M, structure depicted on the right) recorded on a Horiba-JY Fluorolog3 equipped with precisely aligned linear polariser (1:40000, ThorLabs) and a rotatable broad wavelength (λ = 400 – 800 nm) quarter waveplate (QWP, AQWP05M-600, Thorlabs) and linear polariser. This differential CPL spectrum was achieved by recording the left (QWP at 90° to LP) and right (QWP at 90° to LP) spectrum individually. (Black) CPL emission spectra of Λ-Eu:L3 recorded using the gold standard PEM-CPL custom built spectrometer. Both spectra were recorded in MeOH, 295 K, 5 μM complex, λ_{ex} 355 nm, 0.5 nm resolution and 1 ms integration time. The insert picture shows the simplicity of the apparatus (QWP + LP) that is placed perpendicular to the sample holder on the emission arm of the JY Flourolog-3. *(Please note that the extra peeks observed using Fluorlog3-CPL is a result of the combinations of this instrument's higher 0.25 nm resolution (step size) as opposed to the PEN-CPL's 0.5 nm, and its 1.5 nm excitation and emission bandwidth as opposed to 5 nm using PEM-CPL).*

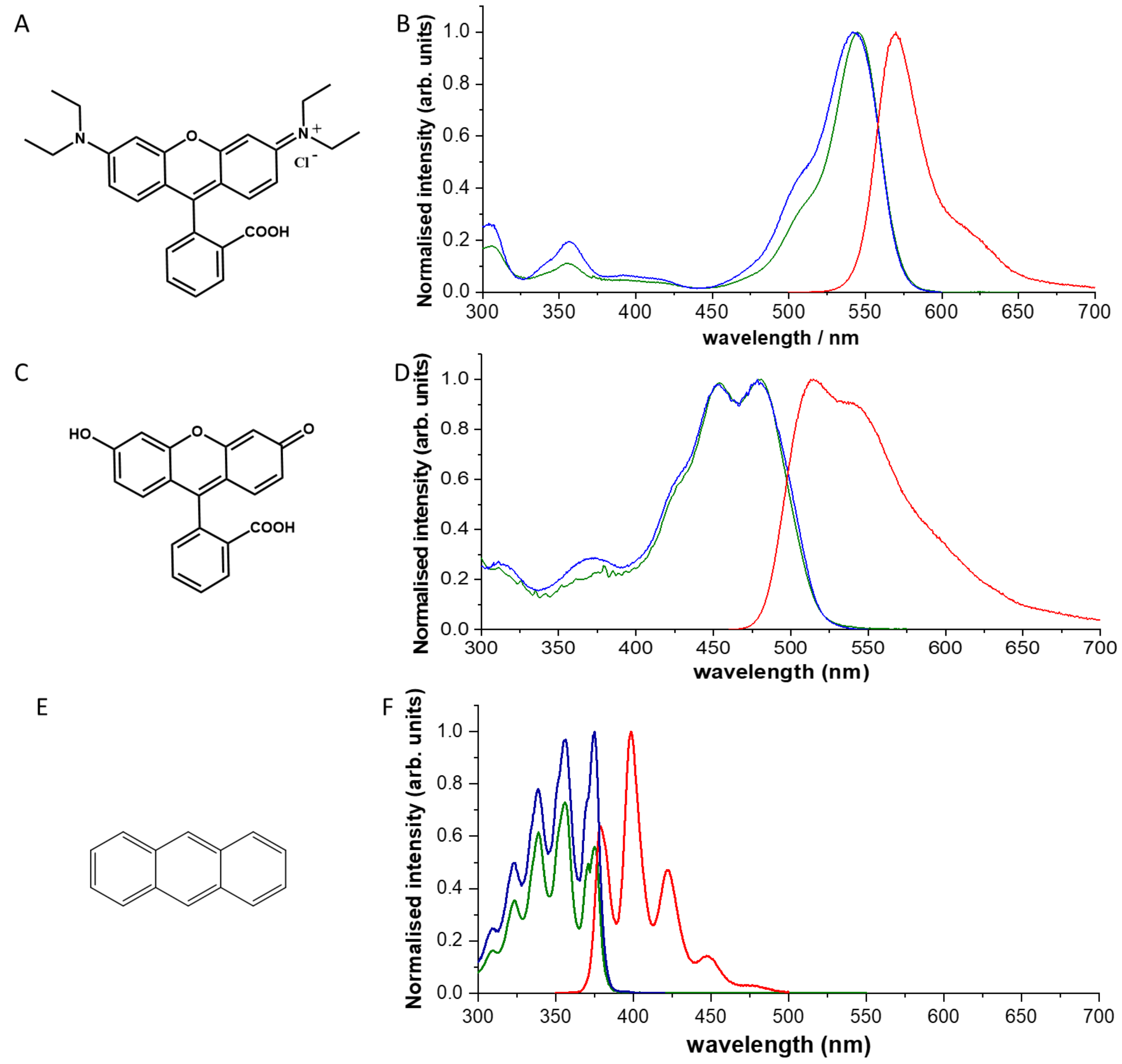

Supplementary Figure 4. Photophysical characteristics of Rhodamine B, Fluorescein and Anthracene used for TR-CPLP. (A) Chemical structure of rhodamine B ($C_{28}H_{31}ClN_2O_3$). (B) Absorption (green), excitation (blue, λ_{em} = 610 nm) and total emission spectra (red, λ_{exc} = 365 nm) of Rhodamine B (C = 2.8 × 10^{-6} M) in EtOAc. (C) Chemical structure of fluorescein ($C_{20}H_{12}O_5$). (D) Absorption (green), excitation (blue, λ_{em} = 514nm) and total emission spectra (red, λ_{exc} = 365 nm) of fluorescein (C = 2.8 × 10^{-6} M) in water. (E) Chemical structure of Anthracene ($C_{14}H_{10}$). (F) Absorption (green), excitation (blue, λ_{em} = 399 nm) and total emission spectra (red, λ_{exc} = 365 nm) of anthracene (C = 2.8 × 10^{-6} M) in EtOH.

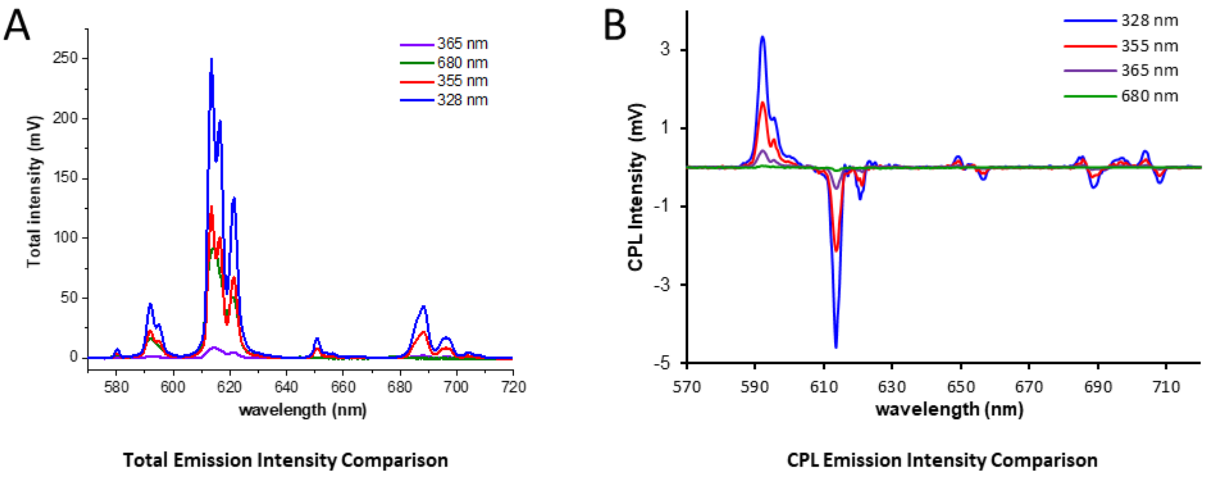

Supplementary Figure 5. Comparison between total emission (A) and CPL spectra (B) of the Λ enantiomer of [Eu:L1] in NMP at various excitation wavelengths ($C = 5 \times 10^{-5}$ M).

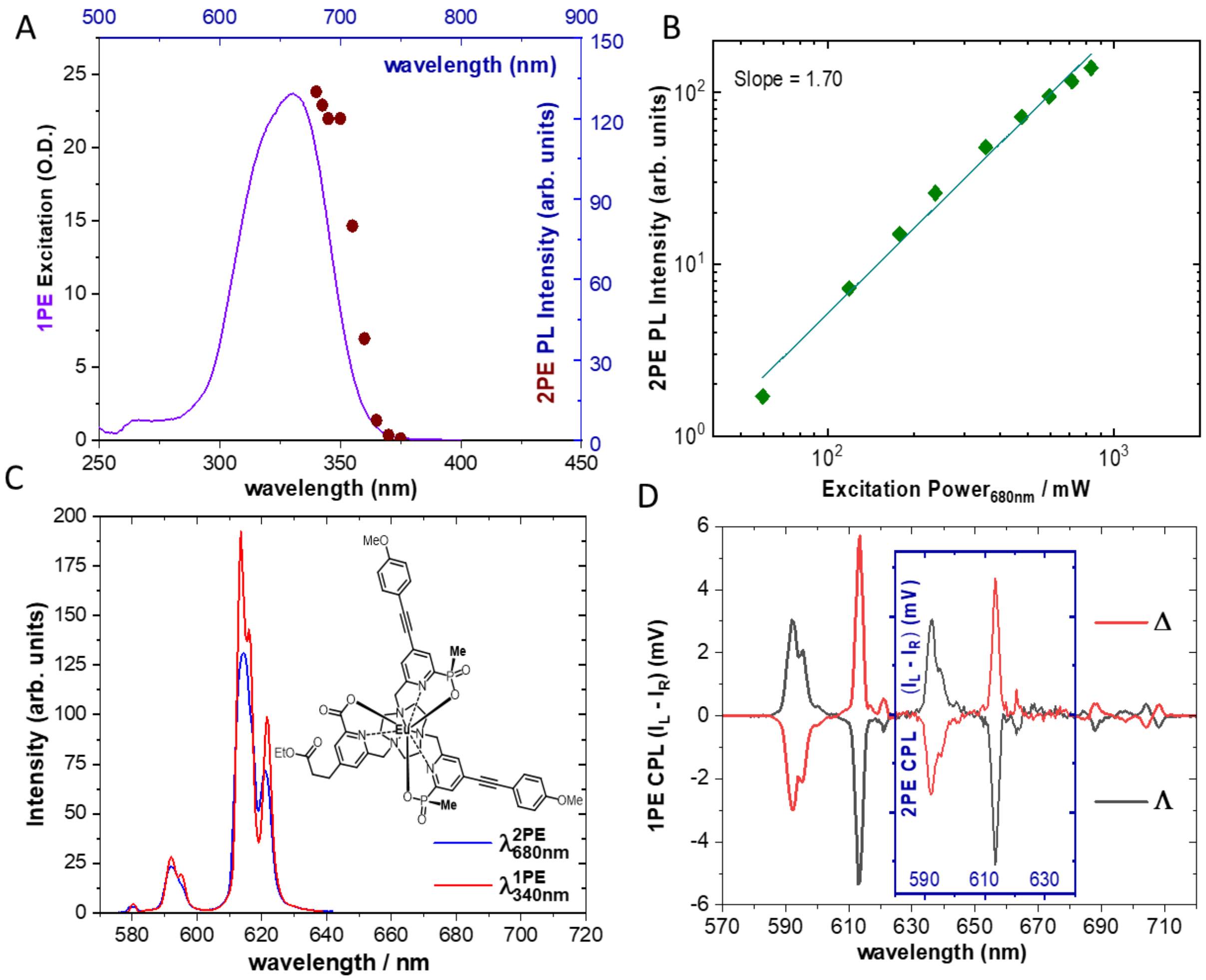

Supplementary Figure 6. Key photophysical parameters and spectra of Λ- and Δ Eu:L1 in EtOAc. (A) One photon excitation (solid purple line) and two photon excitation (maroon dots) spectra (λ_{em} = 614 nm) of Eu:L1. (B) Excitation power dependency (green diamonds) of the 2PE induced photo luminescence (PL) intensity, slope 1.70 ± 0.1, σ^2 = 65 ± 3 GM (10 - 50 cm^4s/photon). (C) One (λ_{ex} = 340 nm, solid red line) and

two photon (λ_{ex} = 680 nm, solid blue line) induced emission spectrum of the depicted (insert) Λ- and Δ Eu:L1. (D) One photon and (insert) two photon CPL spectra of Λ- and Δ Eu:L1 (solid black and red line respectively). Spectra recorded in EtOAc (C = 5 × 10^{-5} M).

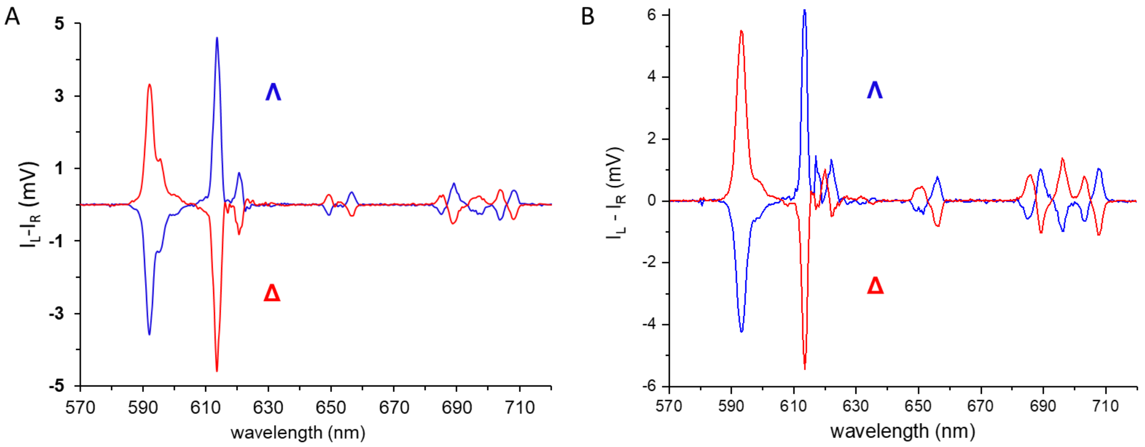

Supplementary Figure 7. CPL spectra of Λ- and Δ Eu:L1 in (A) NMP and in (B) PMMA, C = 5 × 10^{-5} M.

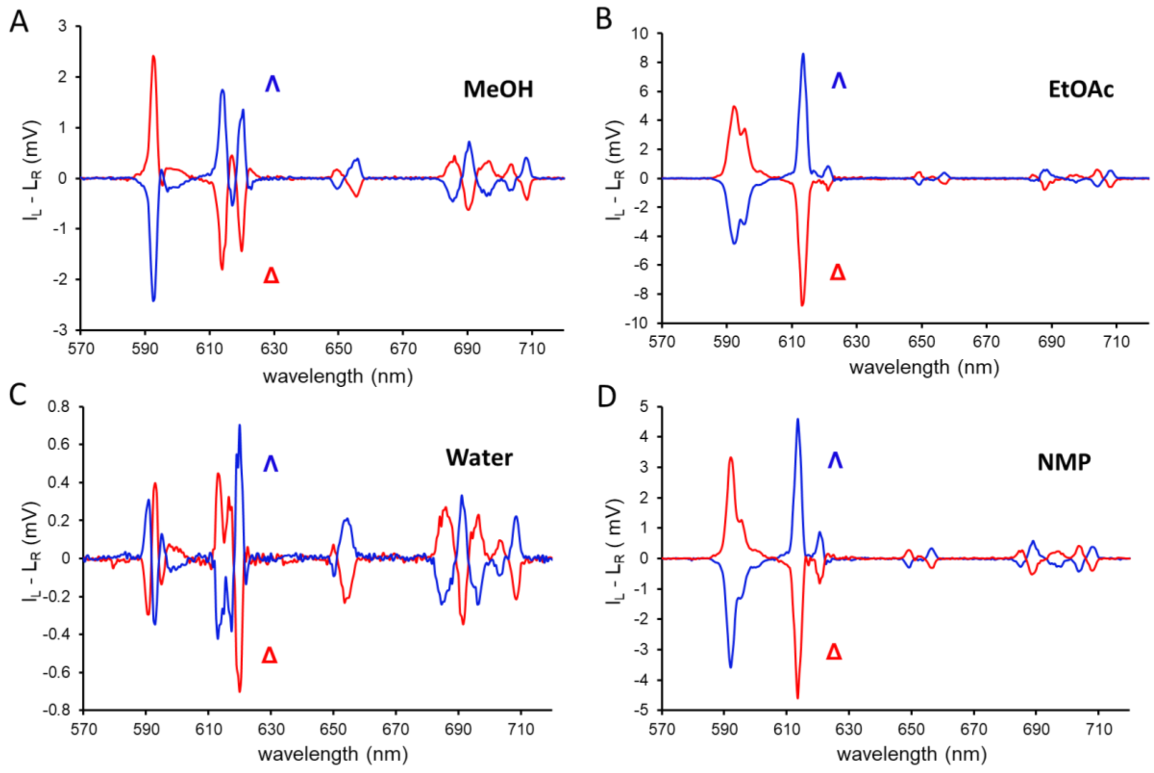

Supplementary Figure 8. CPL spectra of Λ- and Δ Eu:L1 in (A) MeOH, (B) EtOAc, (C) Water and (D) NMP, C = 5 × 10^{-5} M.

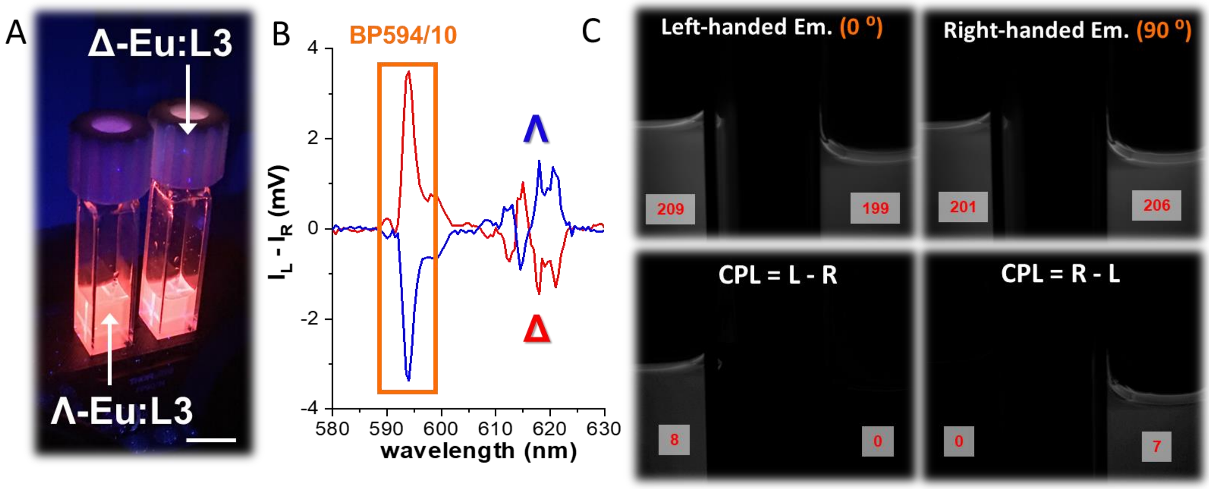

Supplementary Figure 9. Solution state EDCC photography of a CPL active Eu(III) complex. (A) Conventional photo of Λ and Δ-Eu:L3 in MeOH (C = 5 × 10^{-6} M respectively) using 365 nm UV illumination. Scale bar = 1 cm. (B) CPL emission spectra of Δ- (red) and Λ- (blue) enantiomers of Eu:L3 in MeOH (λ_{ex} = 365 nm) highlighting the spectral window selected for photography using an BP594/10 (OD4.0) filter. (C) Images extracted from the quad polarisation view camera highlighting the recorded right- and left-handed emission with respect to the built-in polariser orientation to the fixed QWP fast axis. Using the calculated EDCC images L-R shows clear CPL based emission only for Δ-Eu:L3 whilst R-L shows clear CPL based emission only for Λ-Eu:L3. Numbers in red are avg. 8-bit pixel intensity values for each image region, t_{acq} = 300 ms, 10 avg. image, 100 total image accumulation.

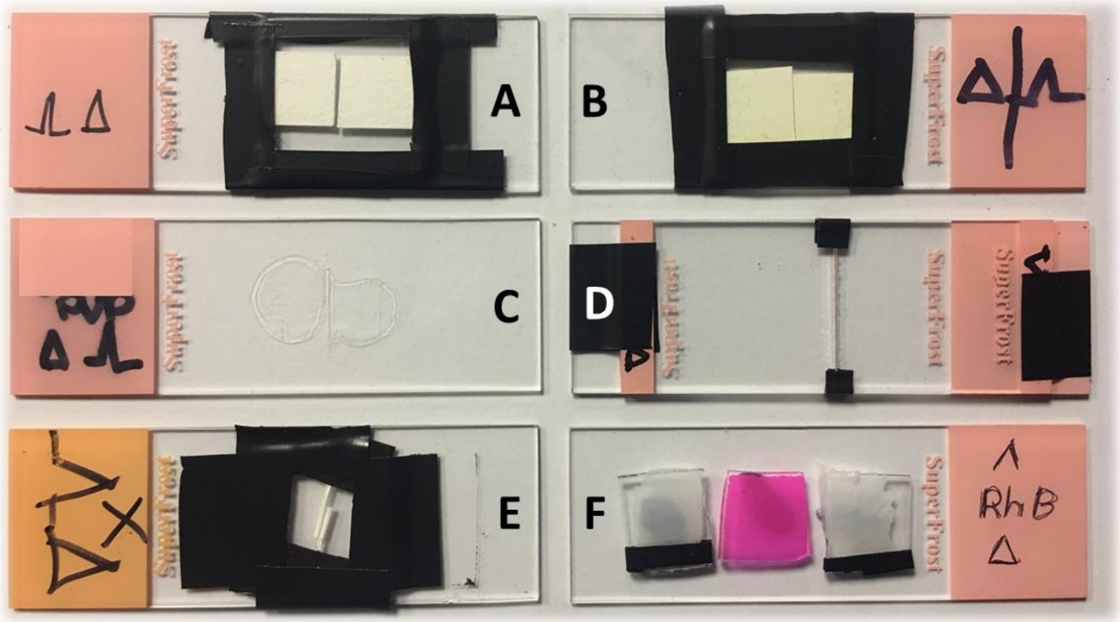

Supplementary Figure 10. Conventional photo of heritage samples on standard microscopy slides containing CPL active Eu(III) complexes. Λ and Δ- Eu:L3 blotted from MeOH onto paper (A) rough side of paper, (B) Smooth side of paper. (C) Λ and Δ- Eu:L3 embedded into a PVP matrix separated by a removable metallic blade to avoid blending. (D) Λ and Δ- Eu:L3 embedded into a PVP matrix on glass surfaces that were cut to size post drying. (E) Λ and Δ- Eu:L4 blotted from MeOH onto optical brightener free photo

paper then cut to size post drying. (F) Λ and Δ- Eu:L1 embedded into a PMMA matrix with cast Rhodamine B PMMA film in-between.

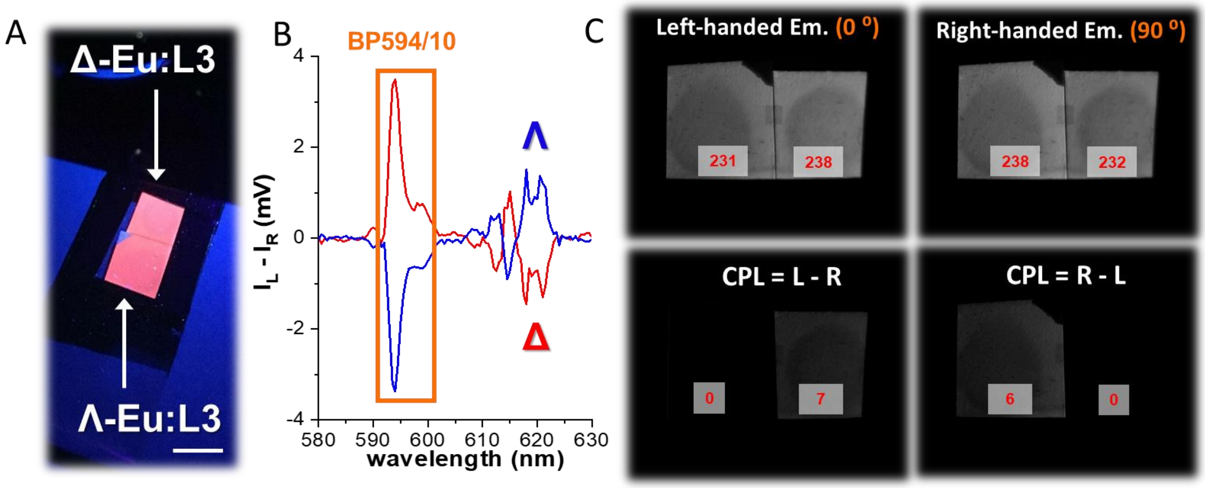

Supplementary Figure 11. Solid state time resolved EDCC photography of a CPL active Eu(III) complex on paper substrate. (A) Conventional photo of Λ and Δ-Eu:L3 in embedded onto optical brightener free paper (C = 5 × 10^{-6} M respectively) using 365 nm UV illumination from the smooth side of the paper. Scale bar = 1 cm. (B) CPL emission spectra of Δ- (red) and Λ- (blue) enantiomers of Eu:L3 in MeOH (λ_{ex} = 365 nm) highlighting the spectral window selected for photography using an BP594/10 (OD4.0) filter. (C) Time resolved (td = 20 μs) Images extracted from the quad polarisation view camera highlighting the recorded right- and left-handed emission with respect to the built-in polariser orientation to the fixed QWP fast axis. Using the calculated EDCC images L-R shows clear CPL based emission only for Δ-Eu:L3 whilst R-L shows clear CPL based emission only for Λ-Eu:L3. Numbers in red are avg. 8-bit pixel intensity values for each image region, t_{acq} = 300 ms, 10 avg. image, 100 total image accumulation.

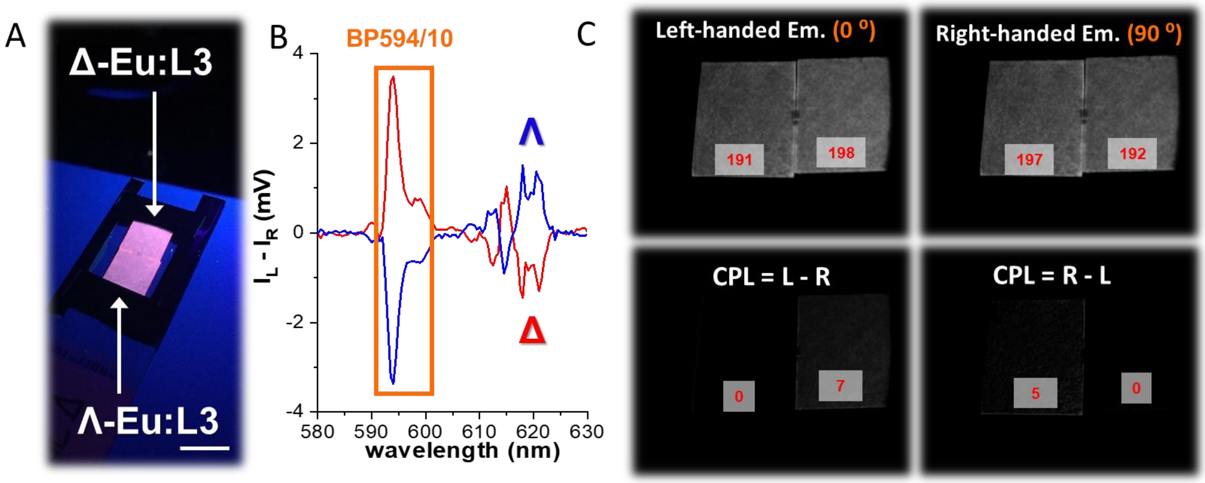

Supplementary Figure 12. Solid state time resolved EDCC photography of a CPL active Eu(III) complex on paper substrate. (A) Conventional photo of Λ and Δ-Eu:L3 in embedded onto optical brightener free paper (C = 5 × 10^{-6} M respectively) using 365 nm UV illumination from the rough side of the paper. Scale bar = 1cm. (B) CPL emission spectra of Δ- (red) and Λ- (blue) enantiomers of Eu:L3 in MeOH (λ_{exc} = 365 nm) highlighting the spectral window selected for photography using an BP594/10 (OD4.0) filter. (C) Time resolved (t_d = 20 μs) Images extracted from the quad polarisation view camera highlighting the recorded right- and left-handed emission with respect to the built-in polariser orientation to the fixed QWP fast

axis. Using the calculated EDCC images: L-R shows clear CPL based emission only for Δ-Eu:L3 whilst R-L shows clear CPL based emission only for Λ-Eu:L3. Numbers in red are avg. 8-bit pixel intensity values for each image region, t_{acq} = 300 ms, 10 avg. image, 100 total image accumulation.

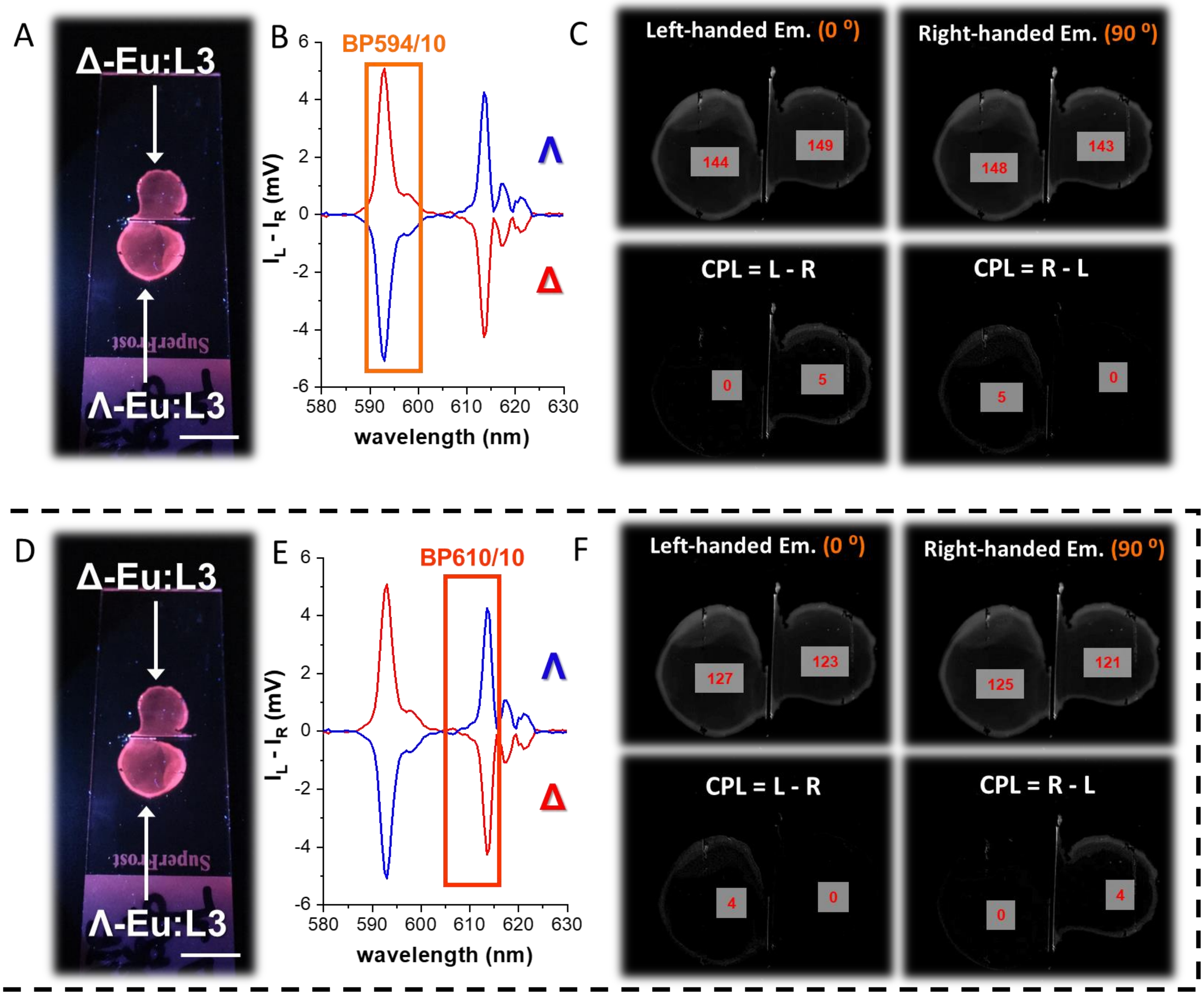

Supplementary Figure 13. Solid state time resolved EDCC photography of a CPL active Eu(III) complex drop cast in PVP. (A) Conventional photo of Λ and Δ-Eu:L3 in embedded into a PVP matrix (C = 5 × 10^{-6} M respectively) using 365 nm UV illumination. Scale bar = 1cm. (B) CPL emission spectra of Δ- (red) and Λ- (blue) enantiomers of Eu:L3 in MNP (λ_{exc} = 365 nm) highlighting the spectral window selected for photography using an BP594/10 (OD4.0) filter. (C) Time resolved (t_d = 20 µs) Images extracted from the quad polarisation view camera highlighting the recorded right- and left-handed emission with respect to the built-in polariser orientation to the fixed QWP fast axis. Using the calculated EDCC images: L-R shows clear CPL based emission only for Δ-Eu:L3 whilst R-L shows clear CPL based emission only for Λ-Eu:L3. Important to note that just as with glass samples any transparent material can lead to solarisation and racemisation of CPL along the peripheral edges as it is evidenced on both L-R and R-L EDCC. Numbers in red are avg. 8 bit pixel intensity values for each image region, t_{acq} = 300 ms, 10 avg. image, 100 total image accumulation. Opposite sign observations were made using BP610/10 (OD4.0), t_{acq} = 700 ms, 10 avg. image, 100 total image accumulation. (Figures D, E and F in dashed black box).

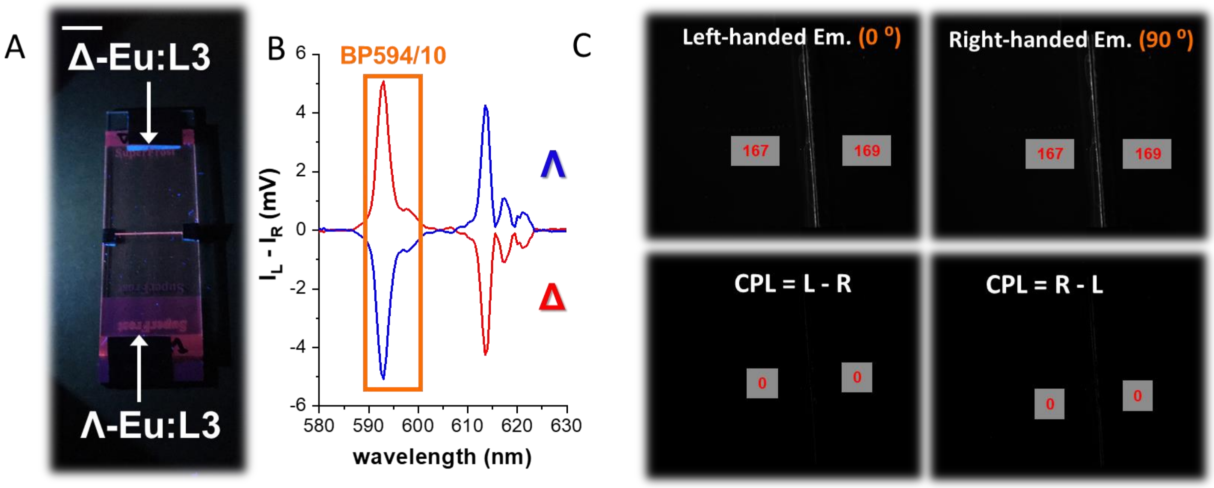

Supplementary Figure 14. Solid state time resolved EDCC photography of a CPL active Eu(III) complex on glass substrate. (A) Conventional photo of Λ and Δ-Eu:L3 in embedded into a PVP matrix on individually cut glass slides ($C = 5 \times 10^{-6}$ M respectively) using 365 nm UV illumination. Scale bar = 1cm. (B) CPL emission spectra of Δ- (red) and Λ- (blue) enantiomers of Eu:L3 in NMP (λ_{exc} = 365 nm) highlighting the spectral window selected for photography using an BP594/10 (OD4.0) filter. (C) Time resolved (t_d = 20 µs) images extracted from the quad polarisation view camera highlighting the recorded right- and left-handed emission with respect to the built-in polariser orientation to the fixed QWP fast axis. EDCC images: No enantioselective imaging can be observed due to reflection (solarisation) induced racemisation of CPL along the glass substrate edge, umbers in red are avg. 8-bit pixel intensity values for each image region, t_{acq} = 300 ms, 10 avg. image, 100 total image accumulation.

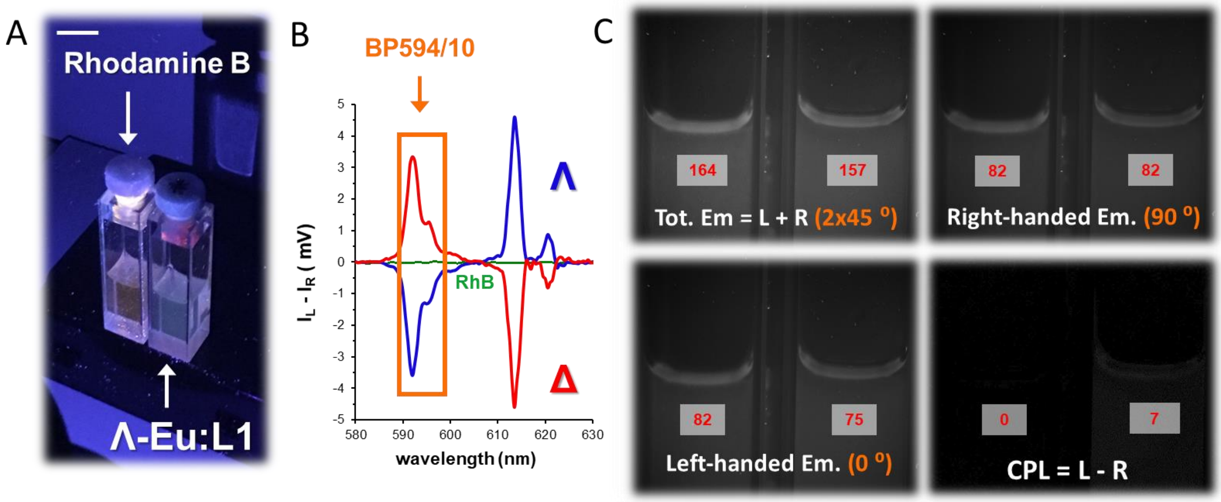

Supplementary Figure 15. **<u>The very first CPLP:</u>** Solution state EDCC photography of an organic emitter and a CPL active Eu(III) complex. (A) Conventional photo of Rhodamine B and Λ-Eu:L1 in EtOAc using 365 nm UV illumination ($C = 3 \times 10^{-6}$ M respectively). Scale bar = 1 cm. (B) CPL emission spectra of (green, Rh B) rhodamine B, Δ- (red) and Λ- (blue) enantiomers of Eu:L1 in EtOAc (λ_{exc} = 365 nm) highlighting the spectral window selected for photography using an BP594/10 (OD4.0) filter. (C) Images extracted from the quad polarisation view camera highlighting the recorded total emission, right- and left-handed emission with respect to the built-in polariser orientation to the fixed QWP fast axis. The calculated EDCC image (R-L) shows clear CPL based emission only for Λ-Eu:L1. Numbers in red are avg. 8-bit pixel intensity values for each image region, $t_{acq.}$ = 250 ms, 10 avg. image, 100 total image accumulation.

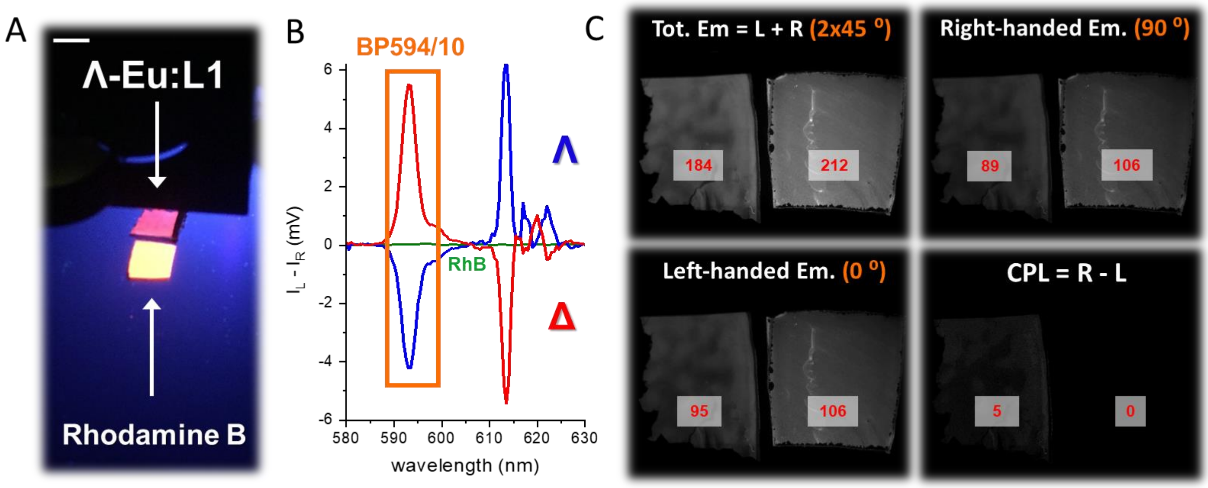

Supplementary Figure 16. Solid state enantioselective differential chiral contrast (EDCC) photography of an organic emitter and a CPL active Eu(III) complex. (A) Conventional photo of Rhodamine B and Λ-Eu:L1 in embedded into a PMMA matrix (C = 3 × 10^{-6} M respectively) using 365 nm UV illumination. Scale bar = 1cm. (B) CPL emission spectra of (green, Rh B) rhodamine B, Δ- (red) and Λ- (blue) enantiomers of Eu:L1 in PMMA film (λ_{exc} = 365 nm) highlighting the spectral window selected for photography using an BP594/10 (OD4.0) filter. (C) Images extracted from the quad polarisation view camera highlighting the recorded total emission, right- and left-handed emission with respect to the built-in polariser orientation to the fixed QWP fast axis. The calculated EDCC image (R-L) shows clear CPL based emission only for Λ-Eu:L1. Numbers in red are avg. 8-bit pixel intensity values for each image region, t_{acq} = 300 ms, 10 avg. image, 100 total image accumulation.

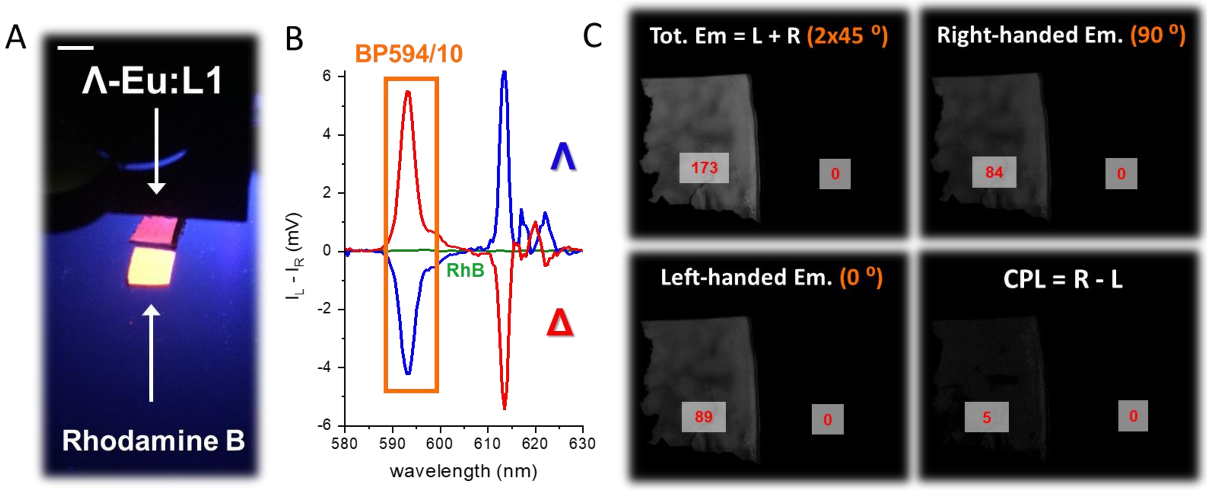

Supplementary Figure 17. Solid state time resolved EDCC photography of an organic emitter and a CPL active Eu(III) complex. (A) Conventional photo of Rhodamine B and Λ-Eu:L1 in embedded into a PMMA matrix (C = 3 × 10^{-6} M respectively) using 365 nm UV illumination. Scale bar = 1cm. (B) CPL emission spectra of (green, RhB) rhodamine B, Δ- (red) and Λ- (blue) enantiomers of Eu:L1 PMMA film (λ_{exc} = 365 nm) highlighting the spectral window selected for photography using an BP594/10 (OD4.0) filter. (C) Time resolved (t_d = 20 μs) Images extracted from the quad polarisation view camera highlighting the recorded total emission, right- and left-handed emission with respect to the built-in polariser orientation to the fixed QWP fast axis. Under time-resolved conditions no emission from the organic fluorophore is detected, whilst the calculated EDCC image (R-L) shows clear CPL based emission only for Λ-Eu:L1.

Numbers in red are avg. 8-bit pixel intensity values for each image region, t_{acq} = 400 ms, 10 avg. image, 100 total image accumulation.

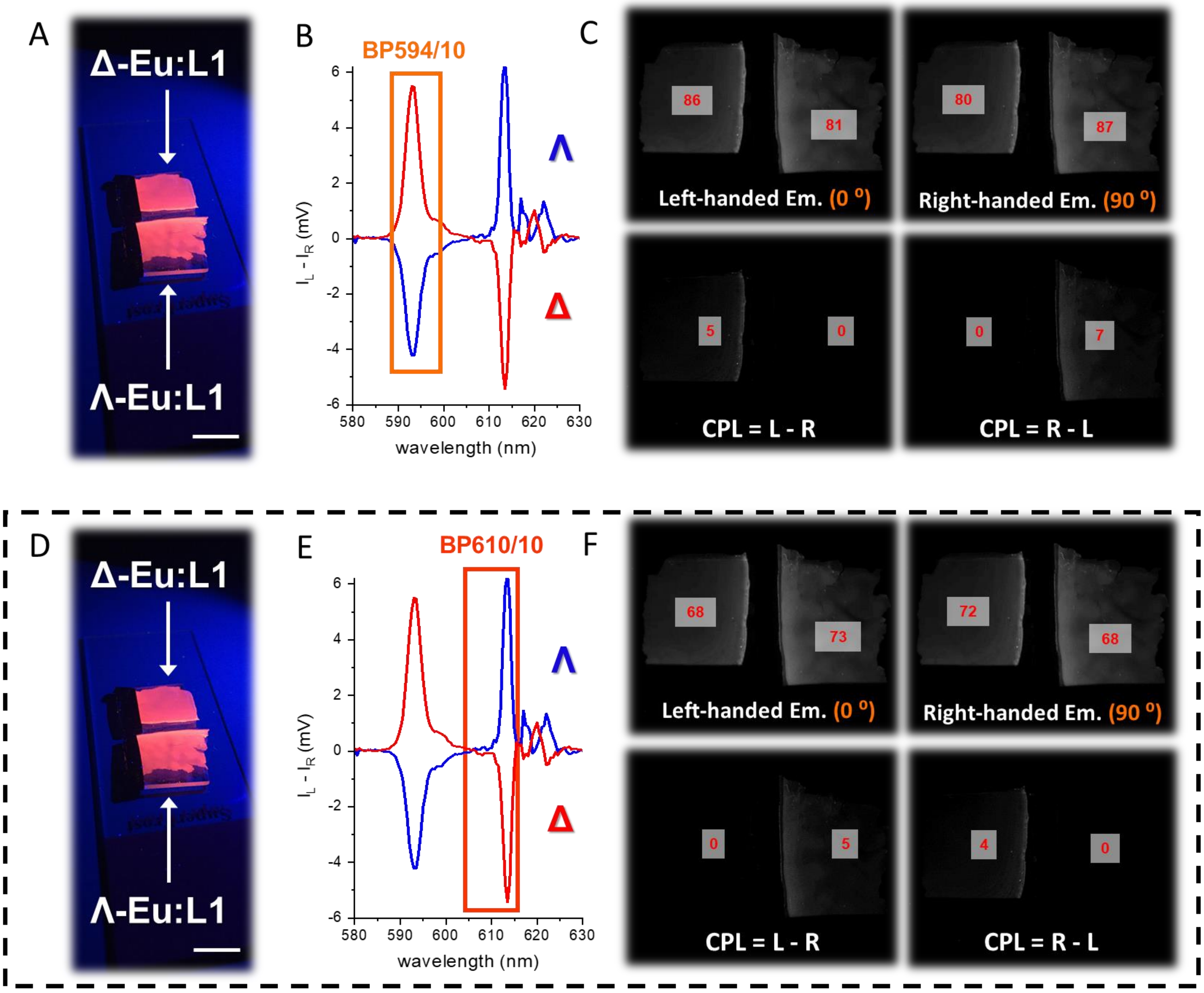

Supplementary Figure 18. Solid state time resolved EDCC photography of an organic emitter and a CPL active Eu(III) complex. (A) Conventional photo of Rhodamine B and Λ-Eu:L1 in embedded into a PMMA matrix (C = 3 × 10^{-6} M respectively) using 365 nm UV illumination. Scale bar = 1 cm. (B) CPL emission spectra of Δ- (red) and Λ- (blue) enantiomers of Eu:L1 in PMMA film (λ_{exc} = 365 nm) highlighting the spectral window selected for photography using an BP594/10 (OD4.0) filter. (C) Time resolved (t_d = 20 µs) Images extracted from the quad polarisation view camera highlighting the recorded total emission, right- and left-handed emission with respect to the built-in polariser orientation to the fixed QWP fast axis. Under time-resolved conditions no emission from the organic fluorophore is detected, whilst the calculated EDCC image (L-R) shows clear CPL based emission only for Δ -Eu:L1 whilst R-L shows clear CPL based emission only for Λ-Eu:L1. Numbers in red are avg. 8-bit pixel intensity values for each image region, t_{acq} = 400 ms, 10 avg. image. Opposite sign observations were made using BP610/10 (OD4.0), t_{acq} = 500 ms, 10 avg. image, 100 total image accumulation (Figures D, E and F in dashed black box).

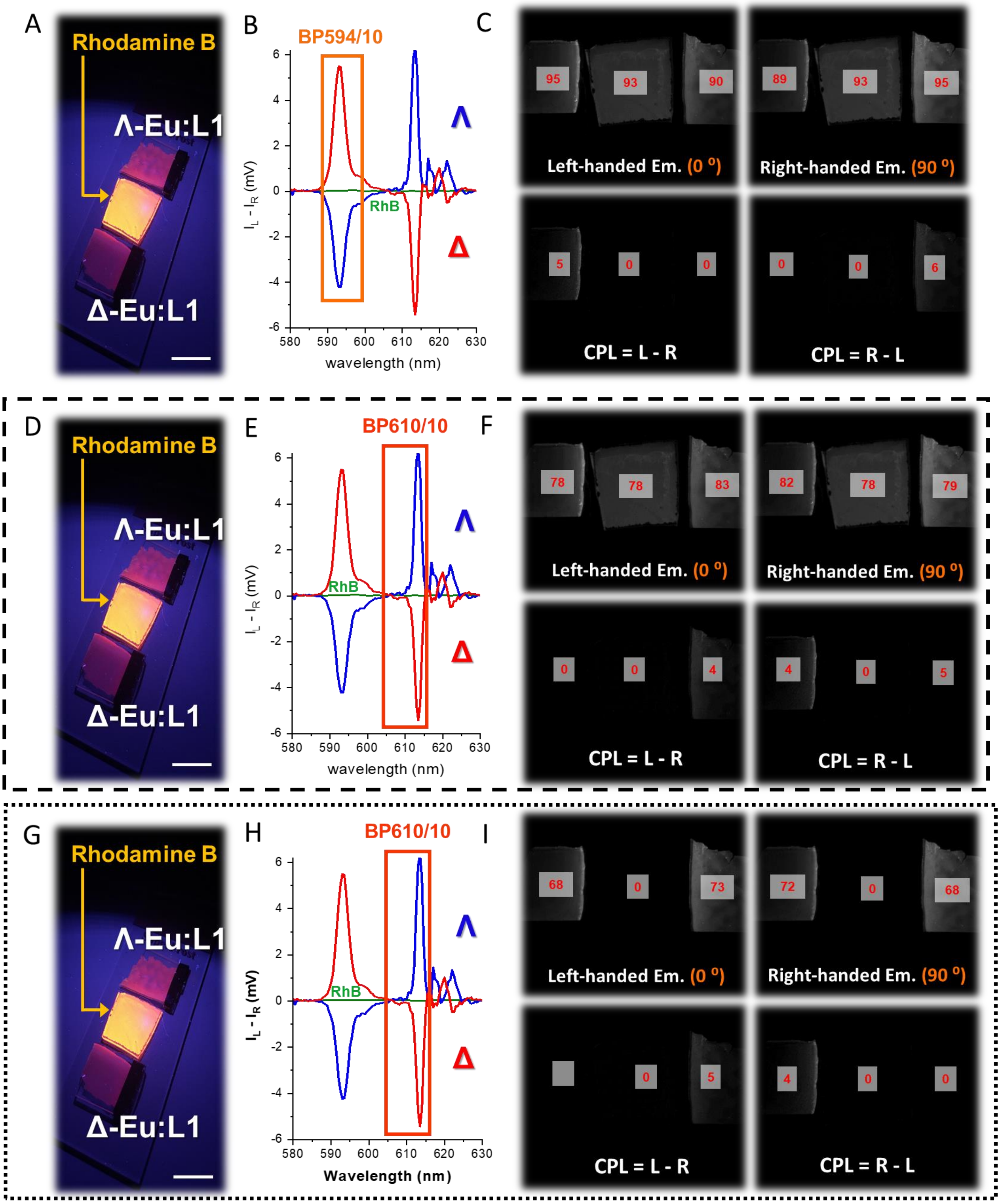

Supplementary Figure 19. Solid state EDCC photography of an organic emitter and a CPL active Eu(III) complex. (A) Conventional photo of Rhodamine B and Λ- and Δ-Eu:L1 in embedded into a PMMA matrix (C = 3 × 10^{-6} M) using 365 nm UV illumination. Scale bar = 1cm. (B) CPL emission spectra of (green, Rh B) rhodamine B, Δ- (red) and Λ- (blue) enantiomers of Eu:L1 in PMMA (λ_{exc} = 365 nm) highlighting the spectral window selected for photography using an BP594/10 (OD4.0) filter. (C) Images extracted from the quad polarisation view camera highlighting the recorded total emission, right- and left-handed emission with respect to the built-in polariser orientation to the fixed QWP fast axis. Using calculated EDCC images: L-R shows clear CPL based emission only for Δ-Eu:L1 whilst R-L shows clear CPL based emission only for Λ-

Eu:L3. Numbers in red are avg. 8-bit pixel intensity values for each image region, t_{acq} = 400 ms, 10 avg. image, 100 total image accumulation. Opposite sign observations were made using BP610/10 (OD4.0) in both steady state (Figure D, E and F in dashed black box) and time-resolved mode (Figures G, H and I in dotted black box), t_{acq} = 700 ms respectively, 10 avg. image, 100 total image accumulation.

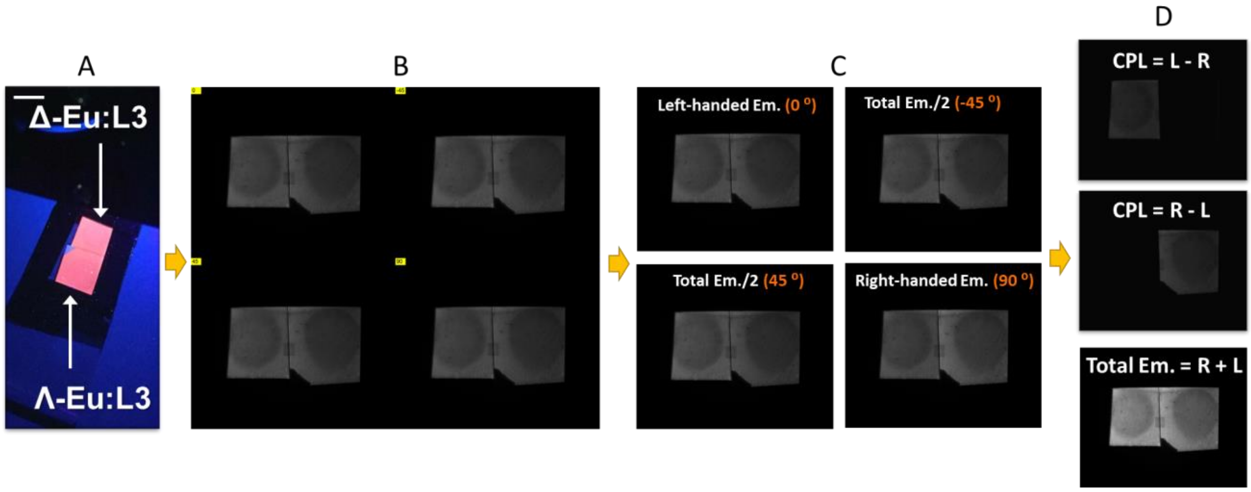

Supplementary Figure 20. CPLP and extraction of EDCC photographic information sequence demonstrating results obtained in Supplementary Figure 11. (A) Conventional photography image. Scale bar = 1cm. (B) Quad-view one shot image of the FOV using ThorCam™ software, 16-bit. (C) Individual 8-bit image generation with respect to built-in wire grid polariser array to extract L-CPL, R-CPL and 2 half intensity total emission image using a custom written macro in ImageJ (v1.49j). (D) EDCC and calculated final image contrast bearing images using ImageJ's built-in calculator plus feature.

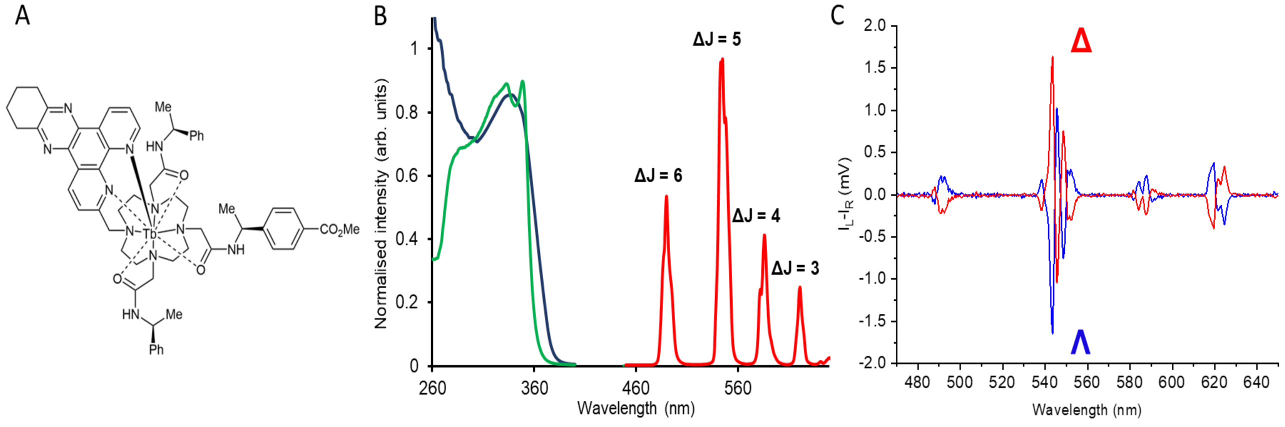

Supplementary Figure 21. Photophysical characteristics of Tb:L6.[2] (A) Chemical structure (B) Absorption (green), excitation (blue, λ_{em} = 544 nm) and total emission spectra (red, λ_{exc} = 344 nm) of Tb:L6 complexes (c = 5.4 x 10^{-5} M) in water. (C) CPL emission spectra of Δ- (red) and Λ- (blue) enantiomers of Tb:L6. Both spectra were recorded in water, 295 K, 5 μM complex, λ_{exc} = 344 nm, 5 scans averaged with 0.5 nm resolution and 1 ms integration time.

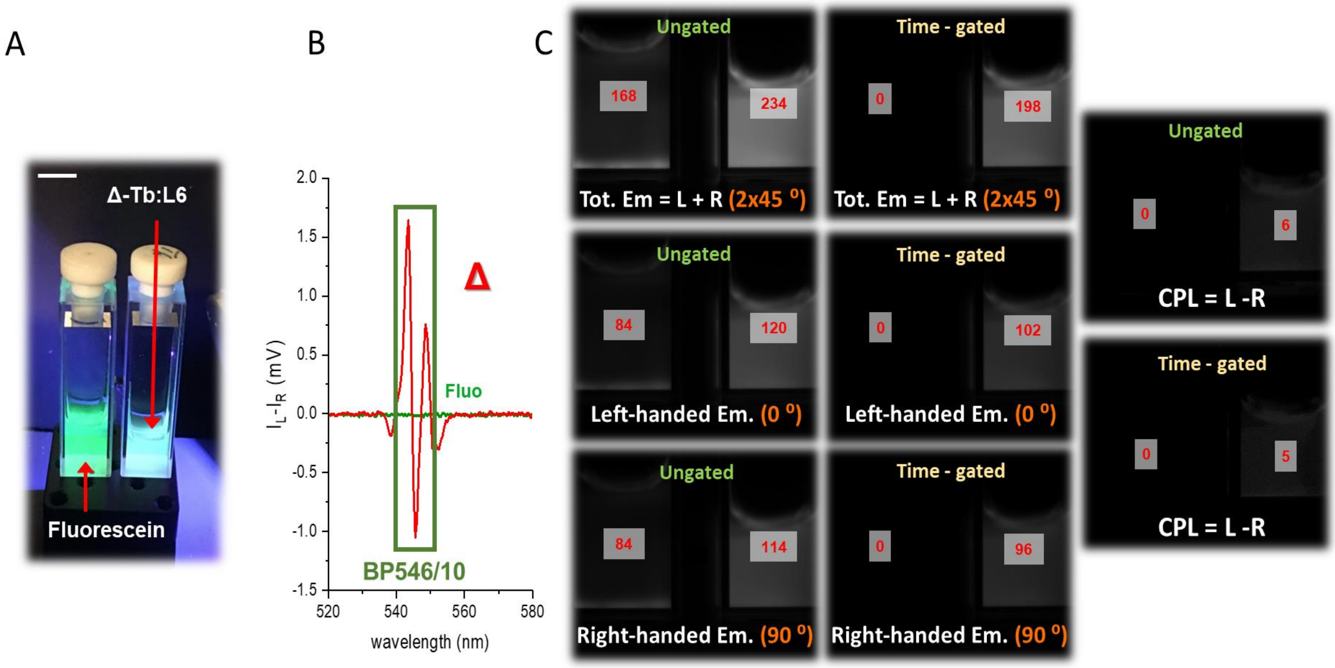

Supplementary Figure 22. Solution state enantioselective differential chiral contrast (EDCC) photography of an organic emitter and a CPL active Tb(III) complex. (A) Conventional photo of fluorescein and Δ-Tb:L6 in water using 365 nm UV illumination (C = 3 × 10^{-6} M respectively). Scale bar = 1cm. (B) CPL emission spectra of (light green, Flu) fluorescein, (green) Δ-enantiomers of Tb:L6 in water (λ_{exc} = 365 nm) highlighting the spectral window selected for photography using an BP546/10 (OD4.0) filter. (C) Images extracted from the quad polarisation view camera highlighting the recorded total emission, right and left-handed emission with respect to the built-in polariser orientation to the fixed QWP fast axis. Time-resolved (t_d = 20 µs) images show no detectable fluorescein emission throughout. The calculated EDCC images (L – R) shows clear CPL based emission only for Δ-Tb:L6 in both ungated and time-gated images. Numbers in red are avg. 8-bit pixel intensity values for each image region, $t_{acq.}$ = 2 s, 10 avg. image, 200 total image accumulation.

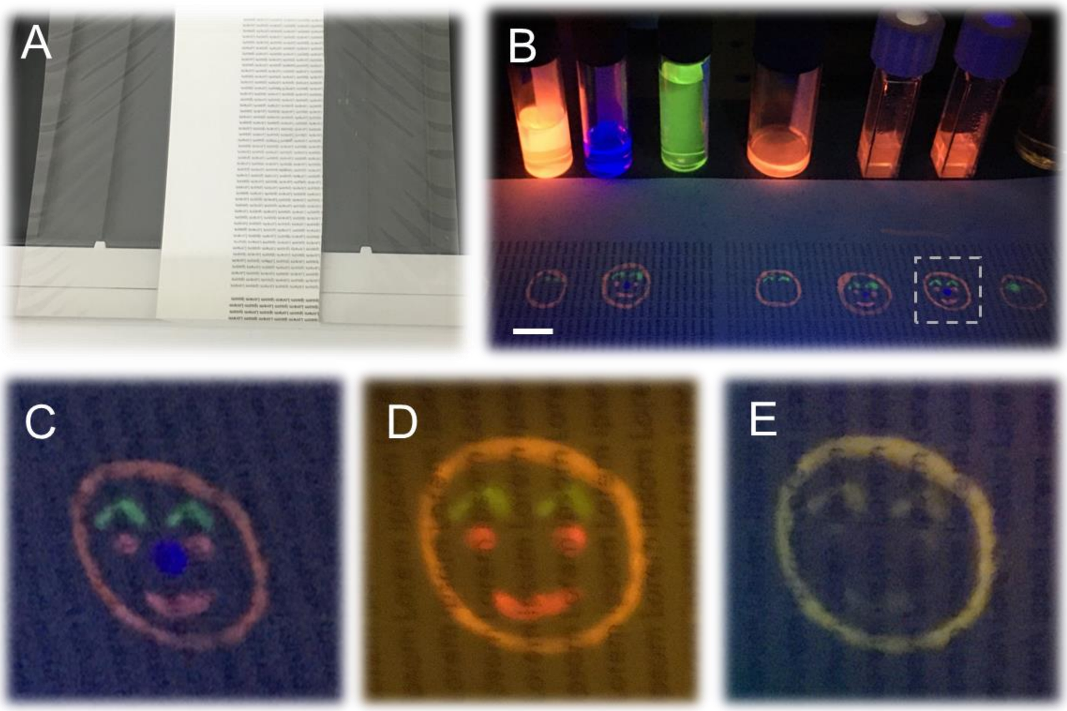

Supplementary Figure 23. Construction and physical composition of the POC CSI security tag (A) Conventional photo of the final laminated (150 ^{o}C) optical brightener-free paper with laser printed text containing the invisible to the naked eye POC CSI security tag. (B) Hand-drawn POC CSI security tags using a calligraphy pen and individual UV (365 nm, standard document reader, LP390 nm emission cut off filter) illuminated solutions of the dyes used (from left to right) Rhodamine B in EtOH, Anthracene in EtOH, Fluorescein in MeOH, Racemate (1:1) Eu:L1, and individual enantiopure Λ-Eu:L1 and Δ-Eu:L1 in EtOAc as a carrier solvent, scale bar 1 cm. (C-E) Zoom-in image of the selected POC CSI tag for CPLP photography imaging under 365 nm UV illumination (laminated, standard UV document reader, iPhone 6S camera) using (C) LP390 nm, (D) LP495 nm and (E) LP570 nm filters.

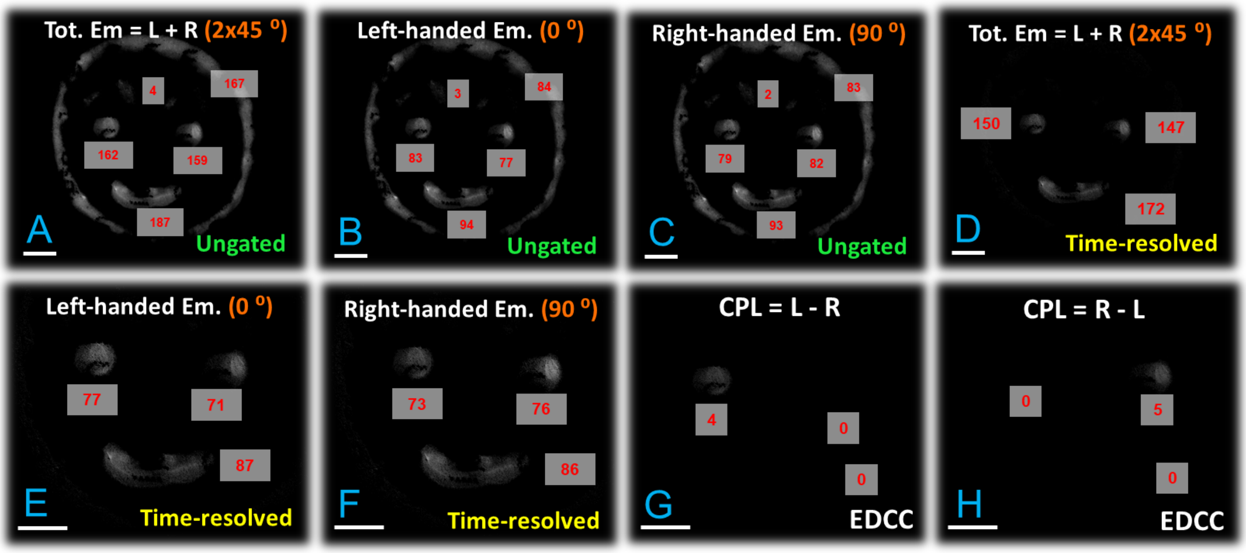

Supplementary Figure 24. Steady state and time-resolved EDCC photography images of the laminated POC CSI security tag. Images extracted (λ_{exc} = 365 nm, λ_{em} = 589-599 nm using a BP594/10 filter) from the quad polarisation view camera highlighting the recorded total emission, right- and left-handed emission with respect to the built-in wire-grid polarised orientation to the fixed QWP fast axis. Total emission under (A) steady state and (D) time-resolved (t_d = 20 µs) conditions. (B) Left- and (C) right-handed emission under steady state condition compared to (E) left- and (F) right-handed emission under time -resolved (t_d = 20 µs) image collection mode. The calculated EDCC image (G) L-R shows clear CPL based emission only for Δ-Eu:L1 whilst (H) R-L shows clear CPL based emission only for Λ-Eu:L1 and demonstrates that EDCC is independent of the applied time domain (steady state vs. time-resolved) mode of collection of CPLP. Numbers in red are avg. 8-bit pixel intensity values for each image region, t_{acq} = 700 ms, 10 avg. image, 10-100 total image accumulation. Scale bar = 2 mm.

Adaptation of CPLP to Laser Scanning Confocal Microscopy (LSCM)

Employing a full frame, full field of view (FOV) detector such as the CPLP camera reported herein, on a scanning excitation beam microscope such as LSCM has its inherent limitation due to prolonged scan times that are incompatible with live cell imaging. Natural cell homeostatic movement occurring while the image is acquired over seconds, resulting in image blurring that is detrimental to both lateral and axial resolution. Moreover, prolonged exposure to excitation light can result in undesirable photobleaching. The LSCM operates by raster scanning the full FOV, it is possible to use the CPLP as a detector and set it

with its longest 14 second exposure time. But this needs to be done by paring this with the Kiralux® camera's maximum full frame averaging sequence of 32 frame (448 seconds in total). In essence this allows the full FOV to be built up from small image segments of 32. Therefore, when the LSCM raster scans the sample in a predetermined X and Y pattern the overall image of the full FOV is built up on the CPLP camera pixel by pixel similarly to a dot matrix printer it builds up an image. However, tailoring this to the CPLP cameras time scale to achieve full FOV forces the user to set the LSCM frame acquisition to match the calculated 448s/frame to record a full FOV. This allows to record a high resolution 2048 x 2048 pixel size frame microscope image (to match the CPLPs native resolution of 2448x2048) at 5 Hz line speed rate. Due to these limitations related to the camera sensitivity, each frame will take a total of 448 second to acquire. With potentially up to 10 images required to be accumulated for a good S/N image, this will further prolong each final image to be acquired over 4480 seconds (just under 75 minutes!). For this reason, the CPLP-LSCM has its obvious limitations for live cell imaging. Each 448 second scan can of course be reduced in 2-bit (2 x 2 = 4) increments by reducing image resolution or increasing LSCM scan speed. This will negatively affect image resolution and overall pixel brightness rendering CPLP-LSCM experimentally limited and challenging for live cell imaging. Nevertheless, as detailed in the main text the incorporation of the CPLP camera as a full field of view detector system into any suitable epifluorescence microscope provides an excellent 'entry level' yet truly versatile microscope capable of EDCC imaging.

Synthesis

Synthesis and characterisation of the legacy complexes **[Eu.L2-5]** and **[Tb.L6]** are described in previous articles from the Parker group.[1,2] Compounds **1**, **5**, and **7** were prepared using previously reported procedures. [1,2]

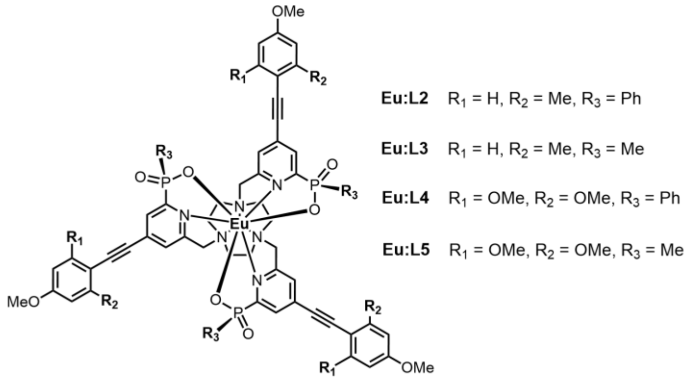

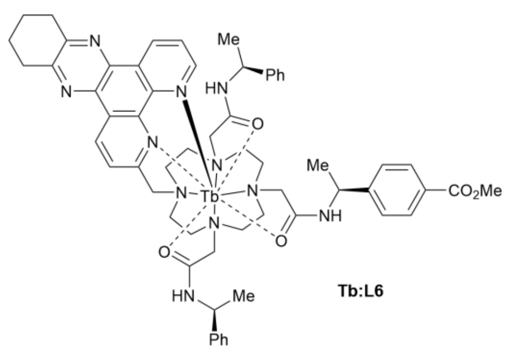

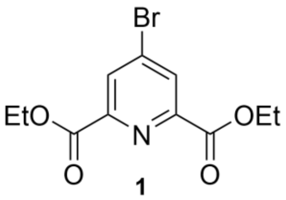
1

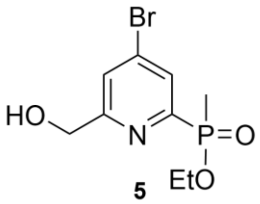
5

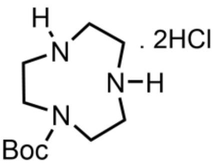
7

Synthesis of pyridyl arm precursor

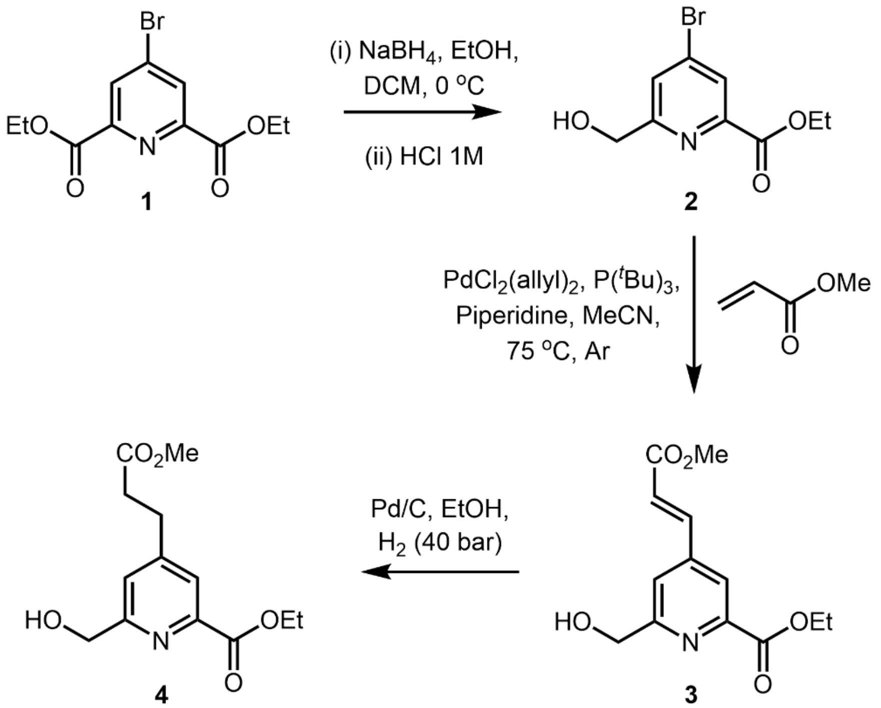

Supplementary Scheme 1. Synthesis of the single pyridyl arm as the alcohol, **4**.

Diethyl 4-bromopyridine-2,6-dicarboxylate, 1

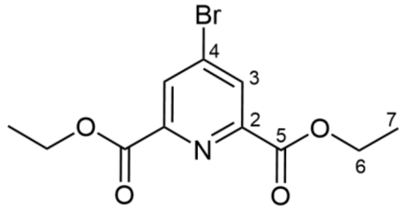

Compound **1** was prepared using established procedures from commercially available chelidamic acid;[11] **^{1}H-NMR** (400 MHz, CDCl$_3$) δ 8.40 (2H, s, H^3), 4.47 (4H, q, $^3J_{H\text{-}H}$ 7, H^6), 1.43 (6H, t, $^3J_{H\text{-}H}$ 7, H^7); **^{13}C-NMR** (101 MHz, CDCl$_3$) δ 163.6 (C^5), 149.5 (C^2), 135.0 (C^4), 131.2 (C^3), 62.8 (C^6), 14.3 (C^7); **ESI-LRMS** (+) *m/z* 302 [M+H]$^+$; **ESI-HRMS** (+) calc. for [$C_{12}H_{13}NO_4Br$]$^+$ 302.0028, found 302.0038.

Ethyl 4-bromopyridine-2-carboxylate, 2

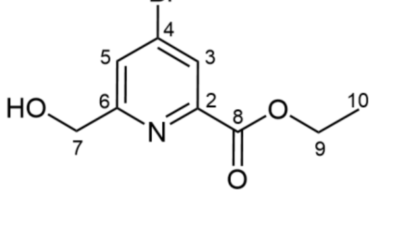

Compound **1** (960 mg, 3.18 mmol) was dissolved in anhydrous DCM (9 mL) and anhydrous EtOH (6 mL). The solution was stirred at 0 °C under argon and sodium borohydride (175 mg, 4.62 mmol) was added slowly. The solution was stirred for 90 min with monitoring by TLC every 15 min (3% MeOH in DCM; R_f 0.3). After 90 min, further sodium borohydride (61 mg, 1.6 mmol) was added to ensure reaction completion. After 3.5 h, the reaction was quenched by slow addition of hydrochloric acid (1 M HCl, 2 mL). The solution was stirred for 10 min before addition of DCM (20 mL) and water (25 mL). The aqueous phase was extracted with DCM (3 × 10 mL), and the combined organic extracts were washed with water (2 × 10 mL), dried over Na_2SO_4 and the solvent evaporated under reduced pressure to yield a yellow oil. The residue was purified by silica column chromatography (0 to 2.5% MeOH in DCM in 0.5% increments) to afford a white solid (489 mg, 59%); **^{1}H-NMR** (600 MHz, $CDCl_3$) δ 8.06 (1H, d, $^4J_{H\text{-}H}$ 2, H^3), 7.74 (1H, d, $^4J_{H\text{-}H}$ 2, H^5), 4.80 (2H, s, H^7), 4.38 (2H, q, $^3J_{H\text{-}H}$ 7, H^9), 4.18 (1H, br s, OH), 1.36 (3H, t, $^3J_{H\text{-}H}$ 7, H^{10}); **^{13}C-NMR** (151 MHz, $CDCl_3$) δ 163.9 (C^8), 162.6 (C^6), 134.5 (C^4), 127.1 (C^5), 126.9 (C^3), 64.4 (C^7), 62.4 (C^9), 14.2 (C^{10}); **ESI-LRMS** (+) *m/z* 260 $[M+H]^+$; **ESI-HRMS** (+) calc. for $[C_9H_{11}NO_3Br]^+$ 259.9922, found 259.9932; MP 63.1 - 66.0 °C.

Ethyl 6-(hydroxymethyl)-4-[3-methoxy-3-oxoprop-1-en-1-yl]pyridine-2-carboxylate, 3

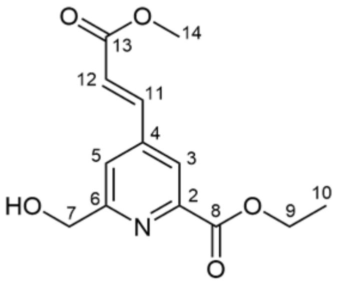

Compound **2** (101 mg, 0.390 mmol) was dissolved in anhydrous acetonitrile (2 mL). Allylpalladium(II) chloride dimer (19 mg, 0.052 mmol), piperidine (0.15 mL, 1.5 mmol), methyl acrylate (0.20 mL, 1.3 mmol), and tri-*tert*-butylphosphine (0.10 mL, 0.42 mmol) were added under argon. The solution was heated at 75 °C for 18 h. The solvents were removed and the residue dissolved in DCM (40 mL) and washed with water (4 × 50 mL). The combined aqueous phases were extracted with DCM (3 × 40 mL). The organic phases were combined, dried over Na_2SO_4, and the solvent evaporated under reduced pressure to afford a dark red oil. The crude residue was purified by reverse-phase HPLC (10 to 100% MeCN in H_2O over 10 min, $\boldsymbol{t_R}$ = 9.0 min) to yield the *E* stereoisomer as a yellow residue (88 mg, 85%); **^{1}H-NMR** (600 MHz, $CDCl_3$) δ 8.08 (1H, s, H^1), 7.64 (1H, d, $^3J_{H\text{-}H}$ 16, H^{11}), 7.59 (1H, s, H^5), 6.68 (1H, d, $^3J_{H\text{-}H}$ 16, H^{12}), 4.88 (2H, s, H^7), 4.47 (2H, q, $^3J_{H\text{-}H}$ 7, H^9), 3.83 (3H, s, H^{14}), 1.43 (3H, t, $^3J_{H\text{-}H}$ 7, H^{10}); **^{13}C-NMR** (151 MHz, $CDCl_3$) δ 166.3 (C^{13}), 164.8 (C^8), 161.6 (C^6), 148.4 (C^2), 143.8 (C^4), 141.1 (C^{11}), 123.8 (C^{12}), 122.0 (C^5), 121.9 (C^3), 64.7 (C^7), 62.3 (C^9),

52.3 (C[14]), 14.4 (C[10]); **ESI-LRMS** (+) *m/z* 266 $[M+H]^+$; **ESI-HRMS** (+) calc. for $[C_{13}H_{16}NO_5]^+$ 266.1028, found 266.1051.

Ethyl 6-(hydroxymethyl)-4-[3-methoxy-3-oxopropyl]pyridine-2-carboxylate, 4

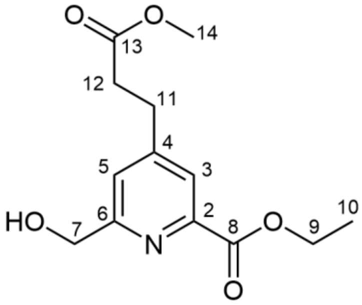

Compound **3** (99 mg, 0.37 mmol) was dissolved in ethanol (20 mL). Palladium on carbon (Pd content 10%, 15 mg) was added and the vessel was loaded onto a Parr hydrogenator (pressure 40 bar H_2) and the reaction mixture was agitated for 4 h. The completion of the hydrogenation reaction was confirmed via LC/MS. The catalyst was removed by filtration and the solvent removed under reduced pressure to yield a white solid (99 mg, quant.); **^{1}H-NMR** (600 MHz, $CDCl_3$) δ 7.83 (1H, s, H[3]), 7.38 (1H, s, H[5]), 4.81 (2H, s, H[7]), 4.43 (2H, q, $^3J_{H-H}$ 7, H[9]), 3.65 (3H, s, H[14]), 3.01 (2H, t, $^3J_{H-H}$ 8, H[11]), 2.67 (2H, t, $^3J_{H-H}$ 8, H[12]), 1.40 (3H, t, $^3J_{H-H}$ 7, H[10]); **^{13}C-NMR** (151 MHz, $CDCl_3$) δ 172.5 (C[13]), 165.2 (C[8]), 160.8 (C[6]), 151.6 (C[4]), 147.6 (C[2]), 123.9 (C[3]), 123.8 (C[5]), 64.6 (C[7]), 62.0 (C[9]), 52.0 (C[14]), 34.1 (C[12]), 30.2 (C[11]), 14.4 (C[10]); **ESI-LRMS** (+) *m/z* 268 $[M+H]^+$; **ESI-HRMS** (+) calc. for $[C_{13}H_{18}NO_5]^+$ 268.1205, found 268.1185.

Synthesis of extended chromophore precursor

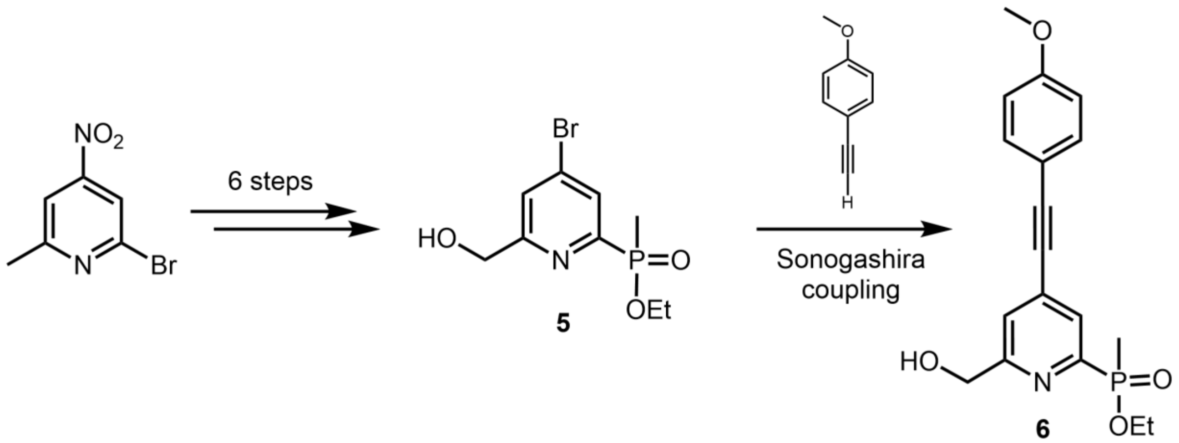

Supplementary Scheme 2. Synthesis of the extended chromophore as the alcohol, **6**. The precursor pyridyl, **5**, was prepared in 6 steps from commercial 2-bromo-6-methyl-4-nitropyridine.[12]

Ethyl (4-bromo-6-hydroxymethylpyridin-2-yl)methylphosphinate, 5

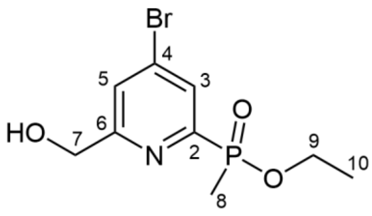

Compound **5** was prepared in six steps from commercially available 2-bromo-6-methyl-4-nitropyridine following a previously reported procedure;[12] **^{1}H-NMR** (400 MHz, $CDCl_3$) δ 8.05 (1H, d, $^{3}J_{H-P}$ 6, H^3), 7.71 (1H, s, H^5), 4.78 (2H, s, H^7), 4.33 (1H, br s, OH), 4.12-3.80 (2H, m, H^9), 1.73 (3H, d, $^{2}J_{H-P}$ 15, H^8), 1.24 (3H, d, $^{3}J_{H-H}$ 7, H^{10}); **^{13}C-NMR** (101 MHz, $CDCl_3$) δ 163.5 (d, $^{3}J_{C-P}$ 20, C^6), 154.7 (d, $^{1}J_{C-P}$ 156, C^2), 134.6 (d, $^{3}J_{C-P}$ 13, C^4), 129.4 (d, $^{2}J_{C-P}$ 22, C^3), 126.5 (d, $^{4}J_{C-P}$ 3, C^5), 64.3 (C^7), 61.8 (d, $^{2}J_{C-P}$ 6, C^9), 16.6 (d, $^{3}J_{C-P}$ 6, C^{10}), 13.8 (d, $^{1}J_{C-P}$ 104, C^8); **ESI-LRMS** (+) *m/z* 294 $[M+H]^+$.

4-((2-(ethoxy(methyl)phosphoryl)-6-(hydroxymethyl)pyridine-4-yl)ethynyl)methoxybenzene, 6

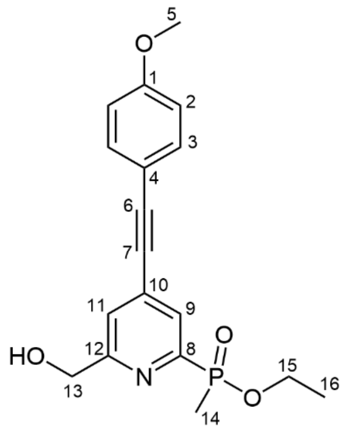

The compound was prepared from compound **5** and commercially available 4-ethynylanisole following established Pd(0)-catalysed Sonogashira cross-coupling methods;[13] **^{1}H-NMR** (400 MHz, $CDCl_3$) δ 8.03 (1H, d, $^{3}J_{H-P}$ 6, H^9), 7.53 (1H, s, H^{11}), 7.44 (2H, d, $^{3}J_{H-H}$ 9, H^2), 6.90 (2H, d, $^{3}J_{H-H}$ 9, H^3), 4.81 (2H, s, H^{13}), 4.17-4.06 (1H, m, H^{15}), 3.92-3.81 (1H, m, H^{15}), 3.80 (3H, s, H^5), 1.78 (3H, d, $^{3}J_{H-P}$ 15, H^{14}), 1.28 (3H, t, $^{4}J_{H-H}$ 7, H^{16}); **^{13}C-NMR** (151 MHz, $CDCl_3$) δ 161.0 (d, $^{3}J_{C-P}$ 19, C^{12}), 160.7 (C^1), 153.3 (d, $^{1}J_{C-P}$ 155, C^8), 133.8 (C^3), 133.2 (d, $^{3}J_{C-P}$ 11, C^{10}), 128.1 (d, $^{2}J_{C-P}$ 22, C^9), 124.2 (d, $^{4}J_{C-P}$ 3, C^{11}), 114.3 (C^2), 113.8 (C^4), 96.3 (C^6), 85.3 (d, C^7), 64.2 (C^{13}), 61.3 (d, $^{2}J_{C-P}$ 6, C^{15}), 55.5 (C^5), 16.5 (d, $^{3}J_{C-P}$ 6, C^{16}), 13.6 (d, $^{1}J_{C-P}$ 107, C^{14}); **ESI-LRMS** (+) *m/z* 347 $[M+H]^+$.

Synthesis of [Eu:L1]

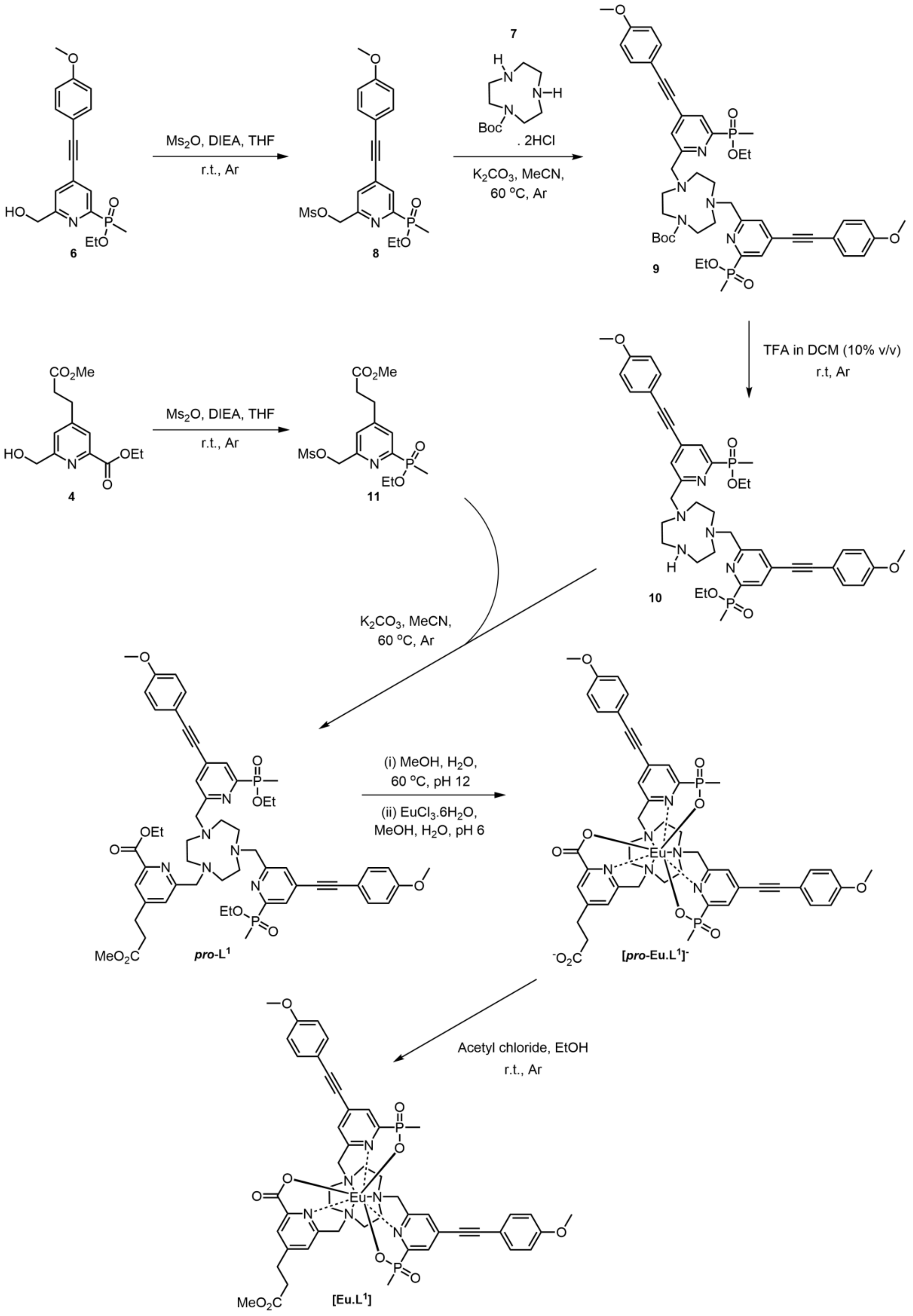

Supplementary Scheme 3. Synthesis of the complex, **[Eu:L1]**. Accurate control on the number of alkylated arms was achieved through Boc protecting group chemistry to afford the pro-ligand, ***pro*-L1**. Subsequent hydrolysis, complexation, and reformation of the methyl ester give the final product, **[Eu:L1]**.

***Tert*-butyl-1,4,7-triazacyclononane-1-carboxylate dihydrochloride, 7**

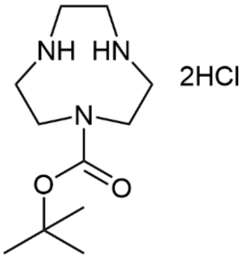

Compound **7** was prepared in three steps from 1,4,7-triazacyclononane following a previously reported procedure.[14]

Compound 8

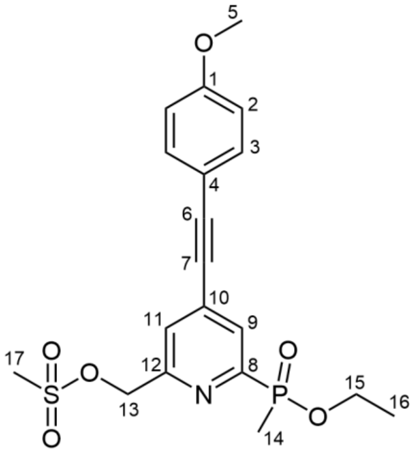

Compound **6** (200 mg, 0.557 mmol) and methanesulfonic anhydride (230 mg, 1.32 mmol) were dissolved in anhydrous THF (3 mL) under argon. DIEA (0.25 mL, 1.4 mmol) was added and the solution stirred for 1 h. The solvents were removed under reduced pressure and the resulting residue was dissolved in DCM (30 mL) and washed with water (3 × 30 mL). The combined aqueous phases were extracted with DCM (3 × 30 mL) and the combined organic phases were dried over Na_2SO_4 and evaporated under reduced pressure to afford a dark yellow oil (243 mg, quant.); **^{1}H-NMR** (400 MHz, $CDCl_3$) *δ* 8.06 (1H, d, $^3J_{H\text{-}P}$ 6, H^9), 7.60 (1H, s, H^{11}), 7.47 (2H, d, $^3J_{H\text{-}H}$ 8, H^3), 6.88 (2H, d, $^4J_{H\text{-}H}$ 2, H^2), 5.35 (2H, s, H^{13}), 4.16-4.05 (1H, m, H^{15}), 3.91-3.82 (1H, m, H^{15}), 3.81 (3H, s, H^5), 3.12 (3H, s, H^{17}), 1.75 (3H, d, $^2J_{H\text{-}P}$ 15, H^{14}), 1.26 (3H, t, $^3J_{H\text{-}H}$ 7, H^{16}); **ESI-LRMS** (+) *m/z* 424 $[M+H]^+$; **ESI-HRMS** (+) calc. for $[C_{19}H_{23}NO_6SP]^+$ 424.0985, found 424.0984.

Compound 9

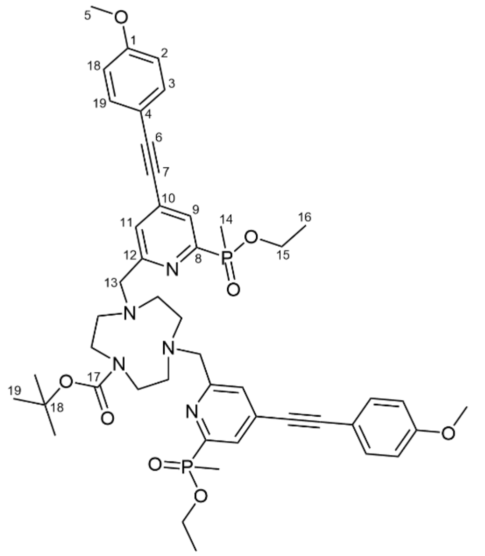

Compound **7** (70 mg, 0.23 mmol) and **8** (240 mg, 0.549 mmol) were dissolved in anhydrous acetonitrile (3 mL) under argon. Potassium carbonate (317 mg, 2.29 mmol) was added and the mixture stirred at 60 °C for 18 h. The reaction solution was separated from the inorganic salts by centrifugation to yield the crude ligand as a yellow residue. The residue was purified via reverse-phase HPLC (10 to 100% MeCN in H_2O over 10 min, $\boldsymbol{t}_{\mathbf{R}}$ = 15.0 min) to yield a dark yellow solid (170 mg, 80%); **^{1}H-NMR** (600 MHz, $CDCl_3$) δ 7.98 (2H, app t, $H^9 + H^{9'}$), 7.67 (1H, s, H^{11}), 7.58 (1H, s, $H^{11'}$), 7.47 (4H, d, $^3J_{H\text{-}H}$ 8, $H^2 + H^{2'}$), 6.89 (4H, d, $^3J_{H\text{-}H}$ 8, $H^3 + H^{3'}$), 4.13-4.05 (2H, m, H^{15}), 3.95 (4H, br, $H^{13} + H^{13'}$), 3.93 (2H, s, $H^{13'}$) 3.90-3.81 (2H, m, $H^{15'}$), 3.83 (6H, s, H^5), 3.42-3.31 (4H, m, 9-N_3 ring), 3.14-3.03 (4H, m, 9-N_3 ring), 2.75-2.62 (4H, m, 9-N_3 ring), 1.76 (6H, 2 × d, $^2J_{H\text{-}P}$ 15, C^{14}), 1.48 (9H, s, H^{19}), 1.25 (6H, 2 × t, $^3J_{H\text{-}H}$ 7, $H^{16} + H^{16'}$); **^{31}P-NMR** (162 MHz, $CDCl_3$) δ +40.2 (2 × s, P); **ESI-LRMS** (+) *m/z* 912 $[M+H]^+$; **ESI-HRMS** (+) calc. for $[C_{49}H_{64}N_5O_8P_2]^+$ 912.4230, found 912.4223.

Compound 10

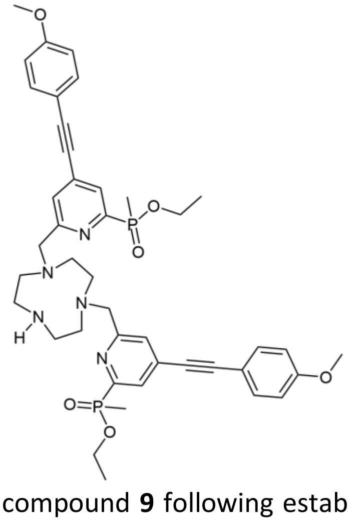

Compound **10** was prepared from compound **9** following established Boc deprotection methods;[13] **ESI-LRMS** (+) *m/z* 912 $[M+H]^+$; **ESI-HRMS** (+) calc. for $[C_{49}H_{64}N_5O_8P_2]^+$ 912.4230, found 912.4223.

Ethyl 6-[(methanesulfonyloxy)methyl]-4-(3-methoxy-3-oxopropyl)pyridine-2-carboxylate, 11

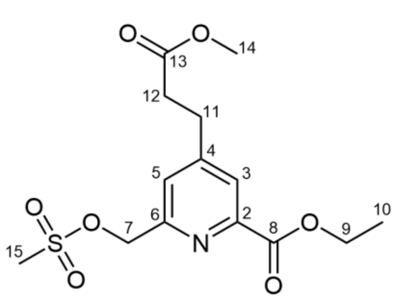

Compound **4** (66 mg, 0.25 mmol) and methanesulfonic anhydride (85 mg, 0.49 mmol) were combined in anhydrous THF (2 mL) under argon. DIEA (0.11 mL, 0.063 mmol) was added and the solution stirred for 1 h. The solvent was evaporated under reduced pressure and the resulting residue dissolved in DCM (20 mL) The organic phase was washed with water (2 × 20 mL) and dried over Na_2SO_4 to yield a dark yellow solid (84 mg, quant.); **^{1}H-NMR** (400 MHz, $CDCl_3$) δ 7.93 (1H, s, H^3), 7.48 (1H, s, H^5), 5.38 (2H, s, H^7), 4.43 (2H, q, $^3J_{H-H}$ 7, H^9), 3.65 (3H, s, H^{14}), 3.15 (3H, s, H^{15}), 3.03 (2H, t, $^3J_{H-H}$ 8, H^{11}), 2.69 (2H, t, $^3J_{H-H}$ 8, H^{12}), 1.39 (3H, t, $^3J_{H-H}$ 7, H^{10}); **ESI-LRMS** (+) *m/z* 346 $[M+H]^+$; **ESI-HRMS** (+) calc. for $[C_{14}H_{20}NO_7S]^+$ 346.0960, found 346.0964.

***pro*-L1**

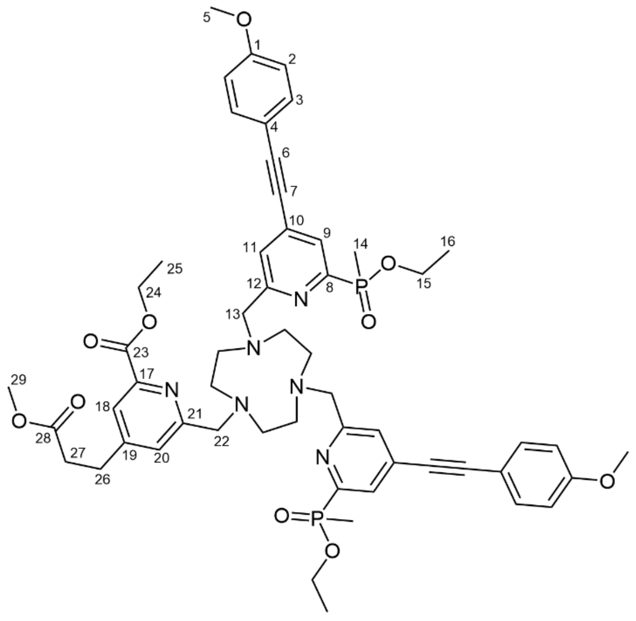

Compound **10** (77 mg, 0.098 mmol), compound **11** (85 mg, 0.25 mmol), and potassium carbonate (40 mg, 0.29 mmol) were combined in anhydrous MeCN (3 mL) under argon. The mixture was stirred at 60 °C for 18 h. The reaction solution was separated from the inorganic salts by centrifugation and concentrated to afford the crude ligand as a yellow residue (58 mg, 57%); **^{1}H-NMR** (400 MHz, $CDCl_3$) δ 7.94 (2H, d, $^5J_{H-P}$ 6, H^{11}), 7.83 (1H, s, H^{18}), 7.81 (2H, d, $^3J_{H-P}$ 11, H^9), 7.44 (4H, d, $^3J_{H\text{-}}$H 9, H^3), 7.39 (1H, s, H^{20}), 6.87 (4H, d, $^3J_{H-H}$ 9,

H[2]), 5.27 (2H, s, H[22]), 4.81 (4H, s, H[13]), 4.42 (2H, q, $^3J_{H\text{-}H}$ 7, H[24]), 4.13-4.02 (2H, m, H[15]), 3.95-3.83 (8H, m, H[15] + 9-N_3 ring), 3.81 (3H, s, H[5]), 3.65 (3H, s, H[29]), 3.00 (2H, t, $^3J_{H\text{-}H}$ 8, H[26]), 2.87 (6H, br s, 9-N_3 ring), 2.67 (2H, t, $^3J_{H\text{-}H}$ 8, H[27]), 1.74 (3H, d, $^2J_{H\text{-}P}$ 16, H[14]), 1.39 (3H, t, $^3J_{H\text{-}H}$ 7, H[25]), 1.22 (6H, t, $^3J_{H\text{-}H}$ 7, H[16]); **ESI-LRMS** (+) *m/z* 1033 $[M+H]^+$; **ESI-HRMS** (+) calc. for $[C_{55}H_{67}N_6O_{10}P_2]^+$ 1033.440, found 1033.444.

[*pro*-Eu:L1]$^-$

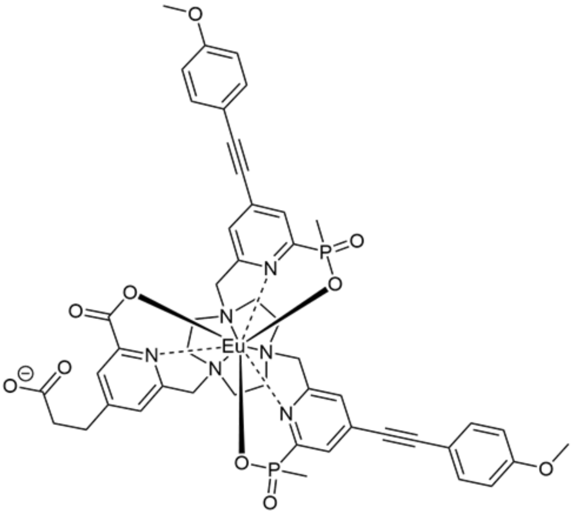

The pro-ligand, ***pro*-L1**, (58 mg) was dissolved in a mixture of MeOH/water (4:1, 3 mL total) and the pH adjusted to 12 using aqueous NaOH solution. The solution was heated at 60 °C for 8 h. The complete hydrolysis of the phosphinate and carboxylic acid ester groups was confirmed by LC/MS analysis. After cooling and adjustment of the pH to 6.5 using aqueous HCl, $EuCl_3 \cdot 6H_2O$ (23 mg, 0.063 mmol) was added. The reaction mixture was heated to 60 °C for 18 h. The solvent was evaporated under reduced pressure to afford a white solid residue. The crude residue was purified by reverse-phase HPLC (10 to 100% MeCN in H_2O over 10 min, $\boldsymbol{t_r}$ = 9.6 min) to yield a white solid (17.5 mg, 29%); **ESI-LRMS** (+) *m/z* 1083 $[M^-+2H]^+$; **ESI-HRMS** (+) calc. for $[C_{48}H_{49}{}^{151}EuN_6O_{10}P_2]^+$ 1083.226, found 1083.229; $\boldsymbol{\tau_{MeOH}}$ (ms) = 1.20, $\boldsymbol{\tau_{H2O}}$ (ms) = 1.00, $\boldsymbol{\tau_{D2O}}$ (ms) = 1.24, $\boldsymbol{\Phi_{MeOH}}$ = 34%, $\boldsymbol{\Phi_{D2O}}$ = 21%.

[Eu:L1]

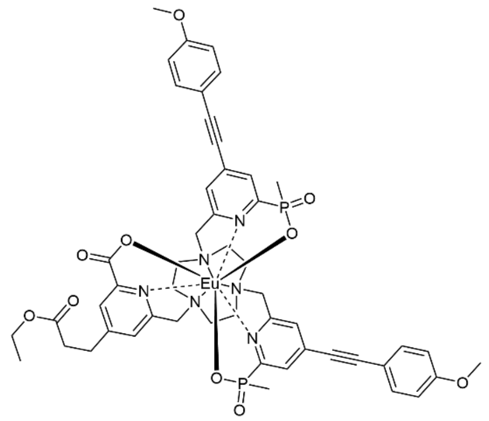

[*pre*-Eu:L1]$^-$ (3.0 mg, 2.8 µmol) was dissolved in anhydrous EtOH (1 mL). A drop of acetyl chloride was added and the mixture stirred under argon at room temperature for 18 h. The solvent was evaporated under reduced pressure to afford a white solid (quant.); Purification and separation of the enantiomers were performed via chiral HPLC, (see Supplementary Figure 25); **ESI-LRMS** (+) *m/z* 1113 $[M+H]^+$; **ESI-HRMS** (+) calc. for $[C_{50}H_{54}{}^{151}EuN_6O_{10}P_2]^+$ 1113.260, found 1113.255.

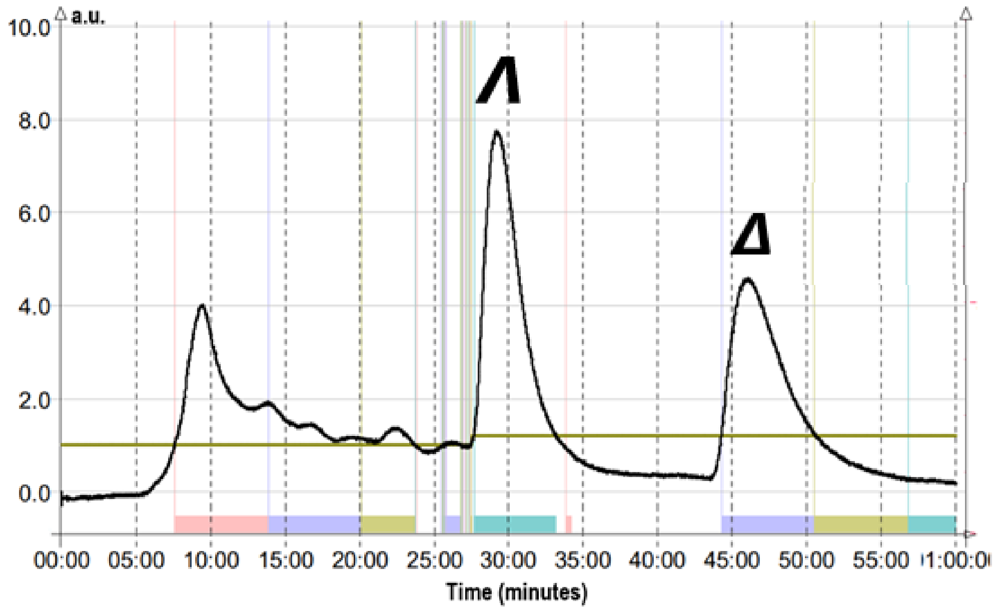

Supplementary Figure 25 Chiral HPLC trace showing the resolution of the enantiomers of Eu:L1 using a CHIRALPAK-IC column (semi-preparative, 295 K, MeOH, 4 mL min^{-1}, UV detector λ = 335 nm). The absolute configuration of the enantiomers was determined by CPL analysis, as previously reported[1] (see Supplementary Figures 4 and 6 for photophysical characterisation).

NMR Spectra

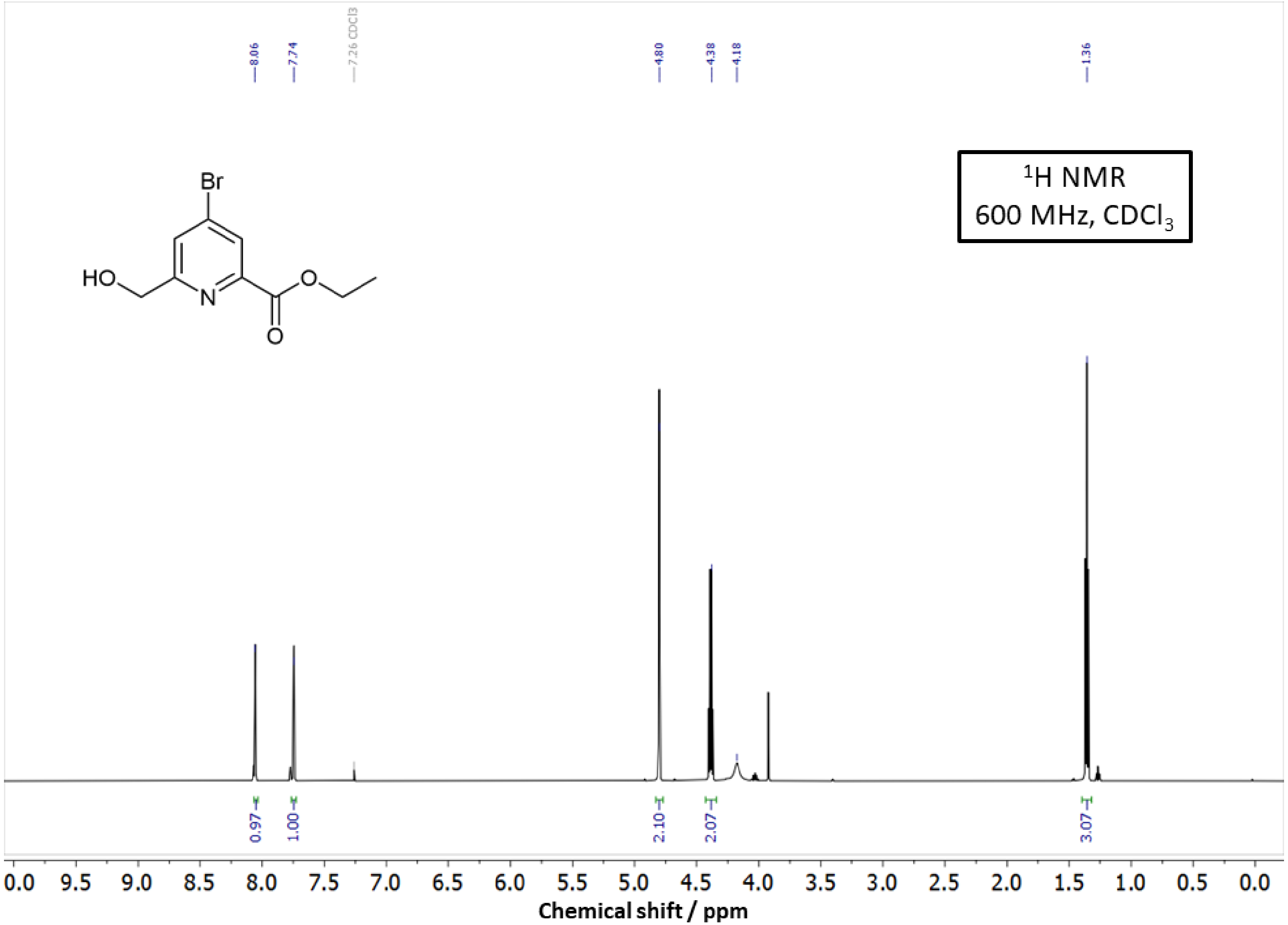

Supplementary Figure 26. ^{1}H NMR (600 MHz, $CDCl_3$) spectrum of compound **2**.

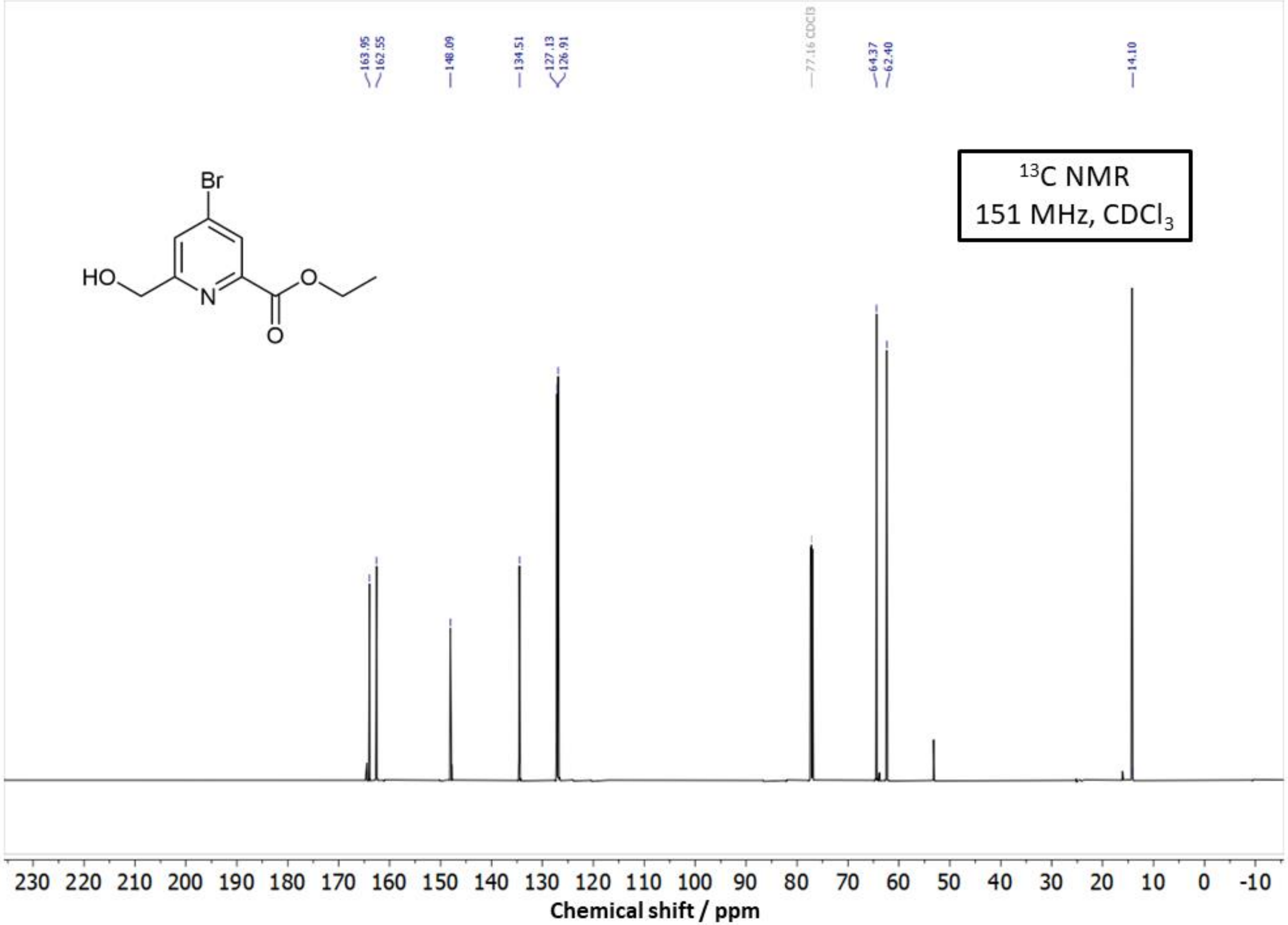

Supplementary Figure 27. ^{13}C NMR (151 MHz, $CDCl_3$) spectrum of compound **2**.

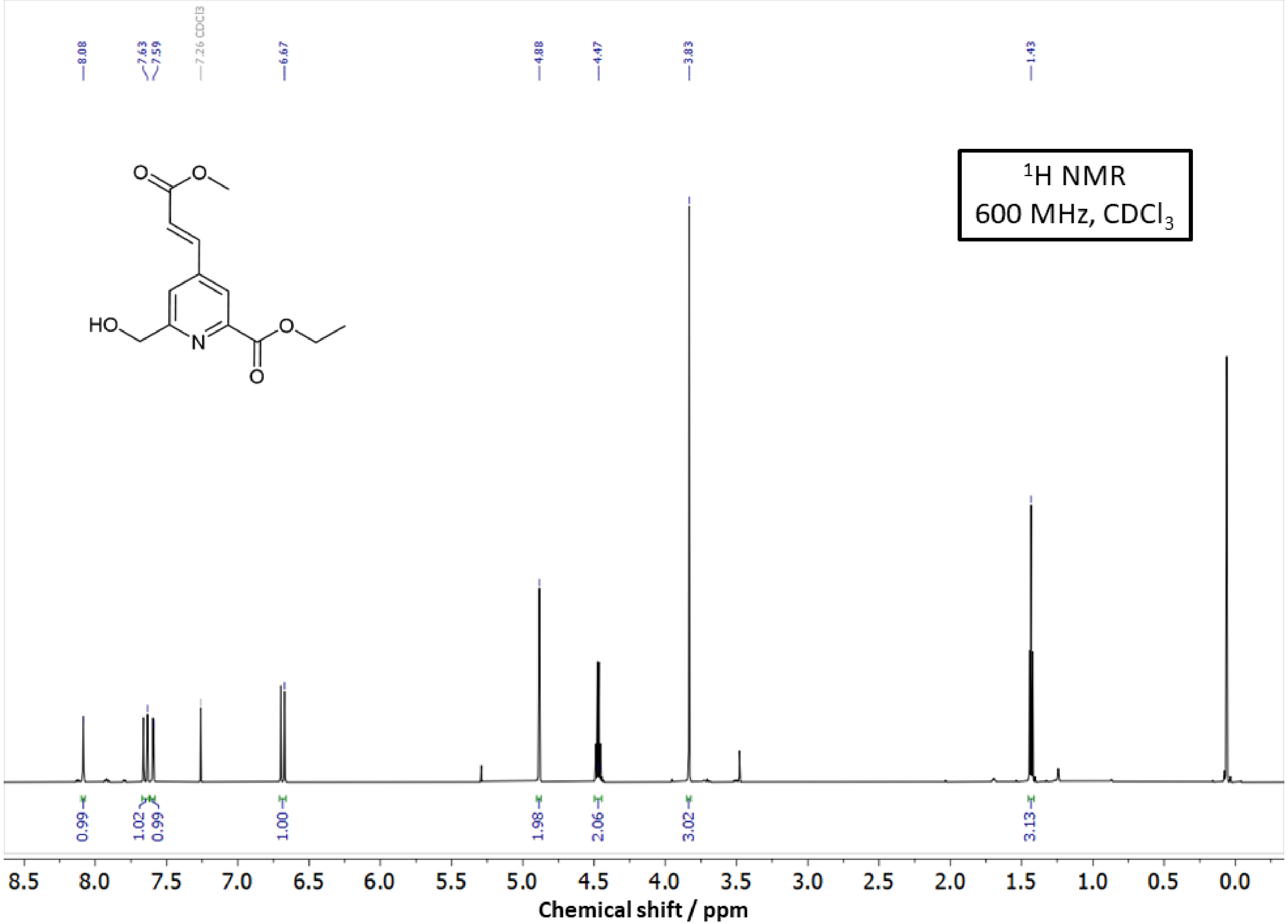

Supplementary Figure 28. ^{1}H NMR (600 MHz, $CDCl_3$) spectrum of compound **3**.

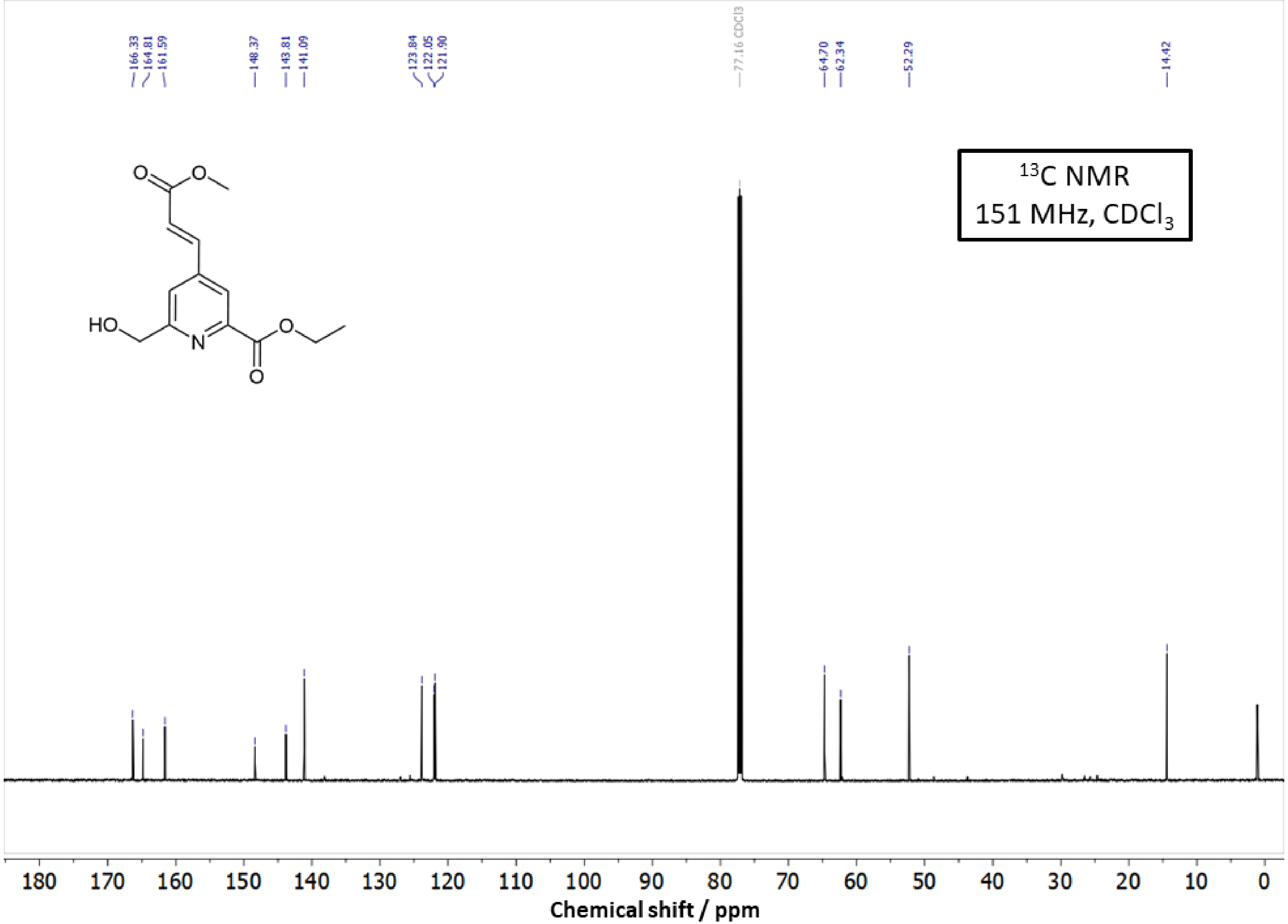

Supplementary Figure 29. ^{13}C NMR (151 MHz, $CDCl_3$) spectrum of compound **3**.

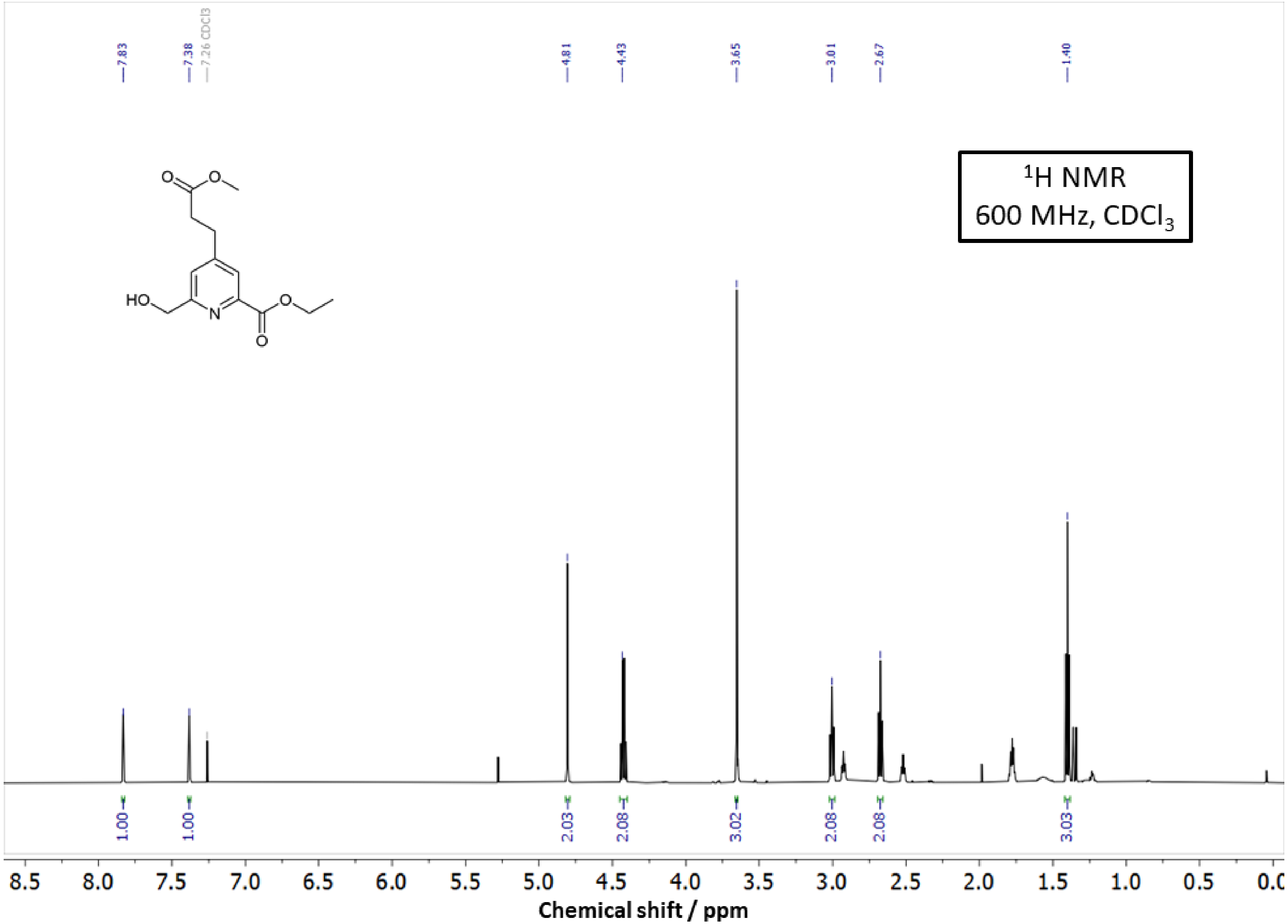

Supplementary Figure 30. ^{1}H NMR (600 MHz, $CDCl_3$) spectrum of compound **4**.

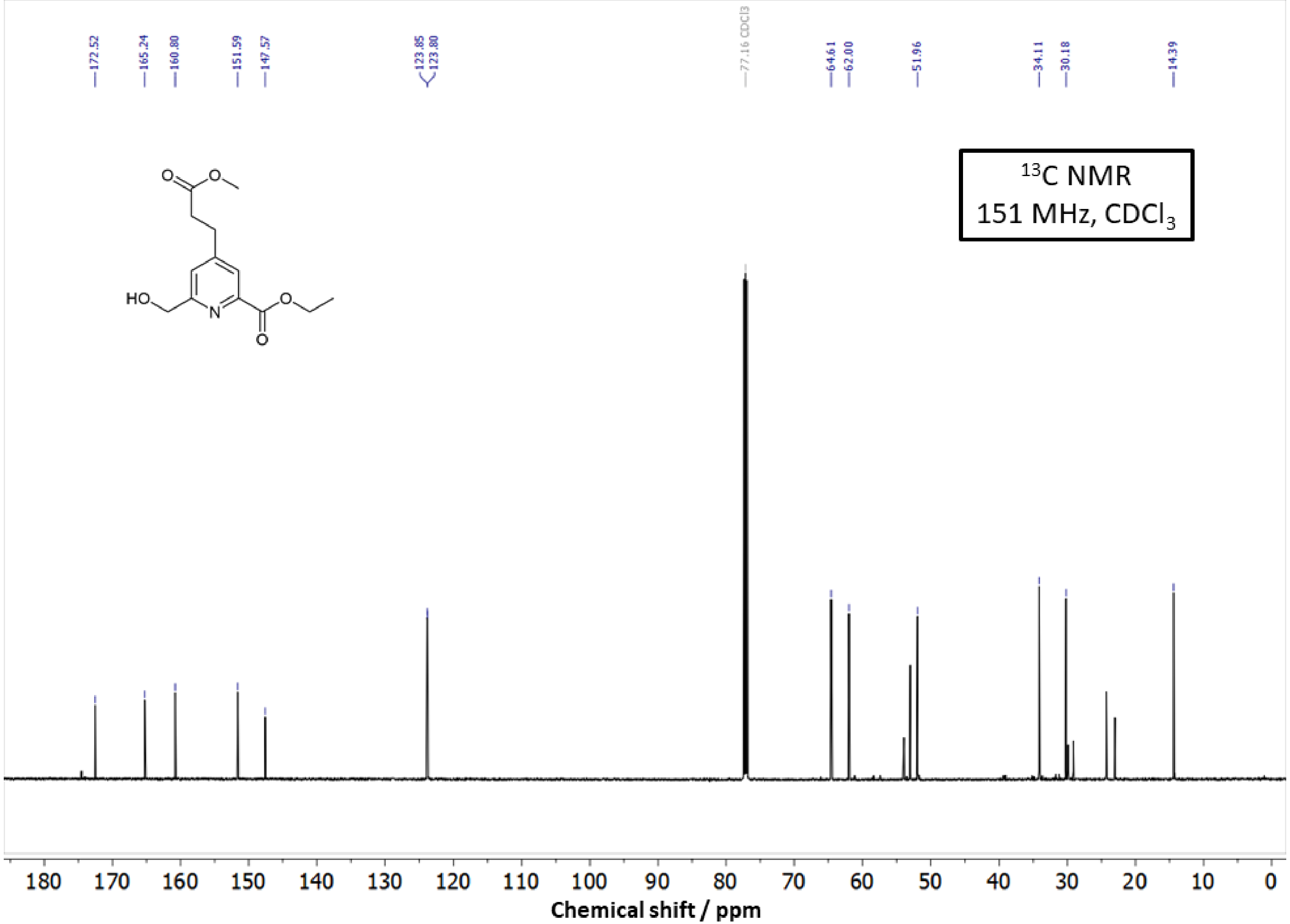

Supplementary Figure 31. ^{13}C NMR (151 MHz, $CDCl_3$) spectrum of compound **4**.

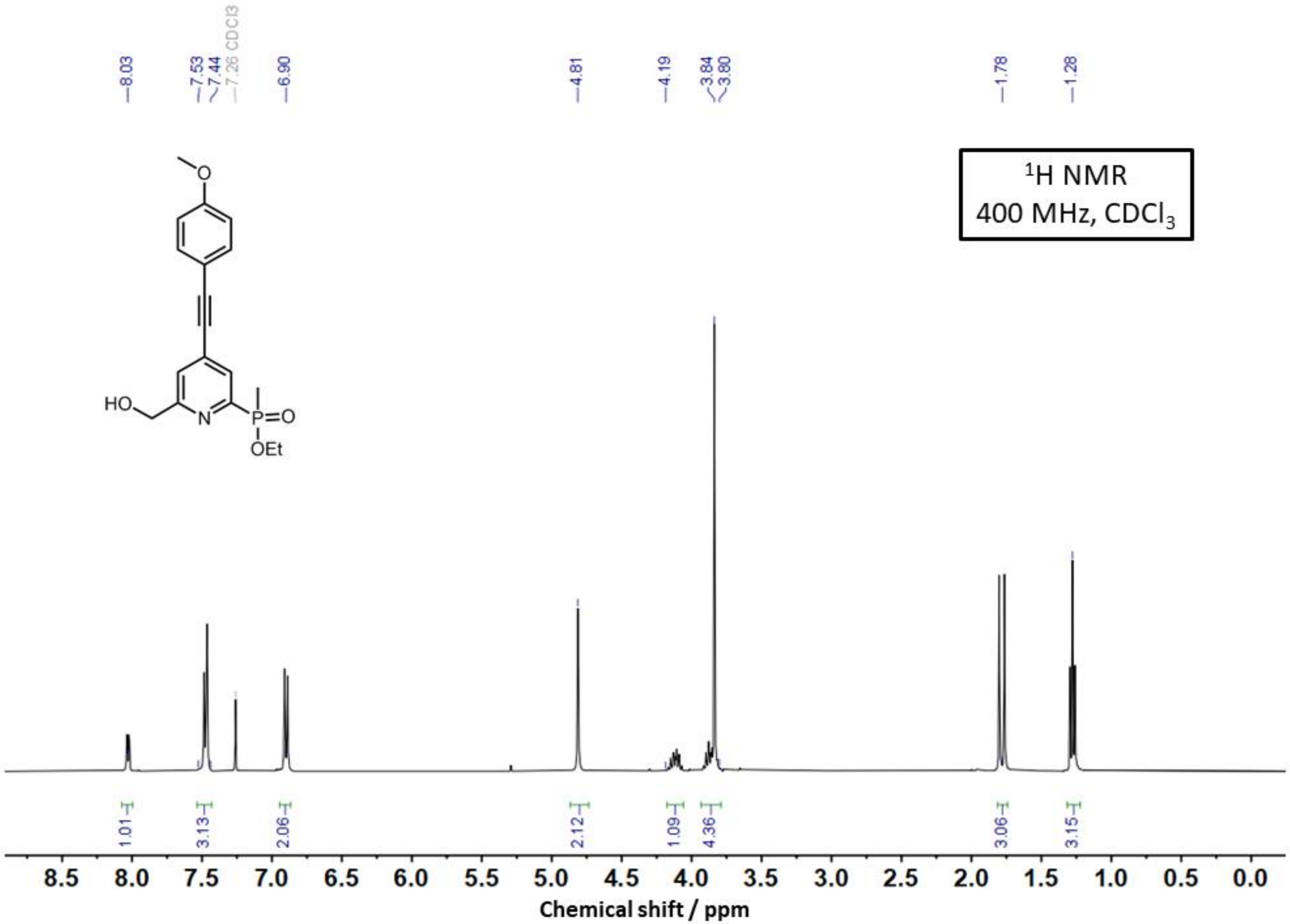

Supplementary Figure 32. ^{1}H NMR (600 MHz, $CDCl_3$) spectrum of compound **6**.

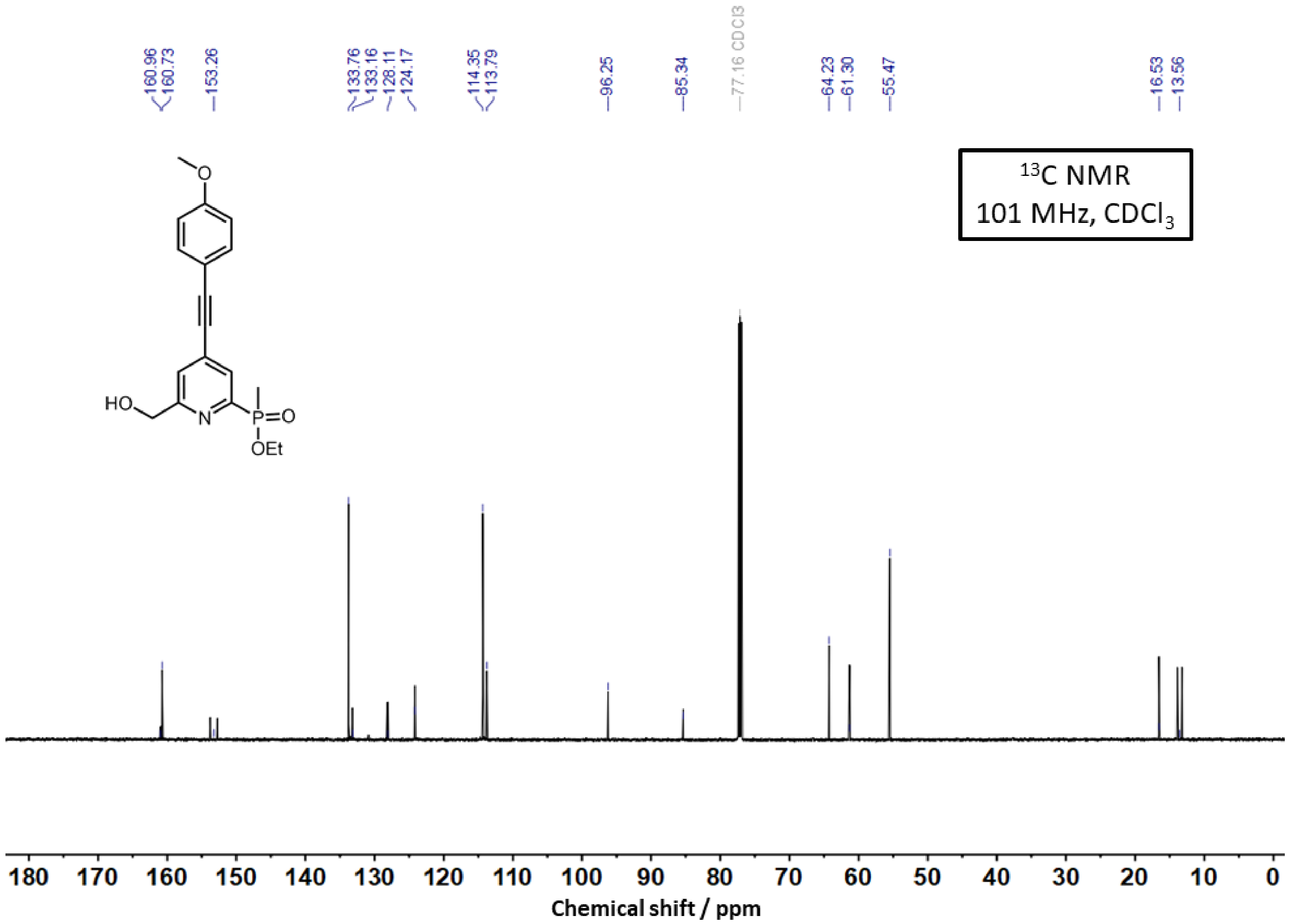

Supplementary Figure 33. ^{13}C NMR (101 MHz, $CDCl_3$) spectrum of compound **6**.

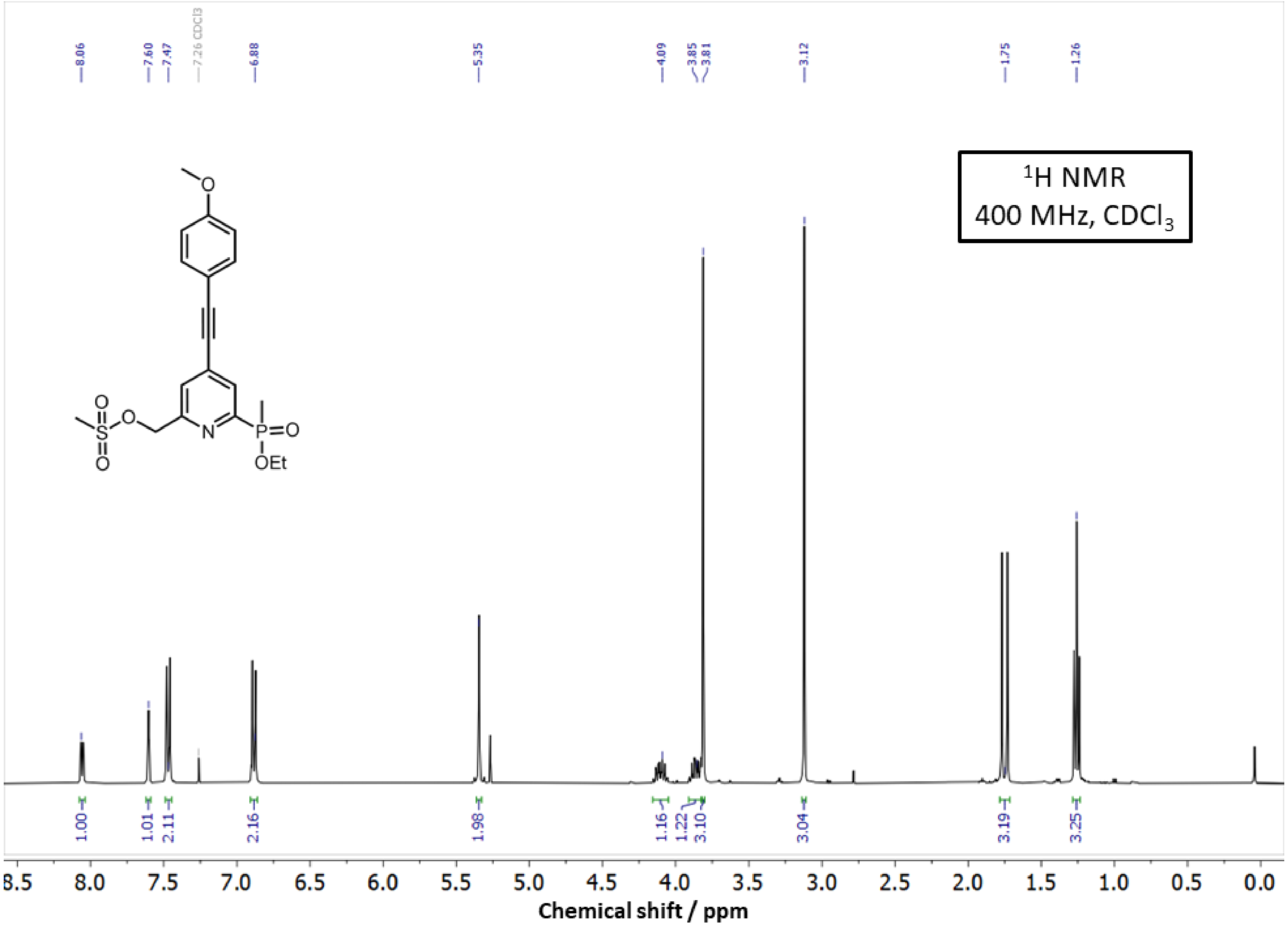

Supplementary Figure 34. ^{1}H NMR (400 MHz, $CDCl_3$) spectrum of compound **8**.

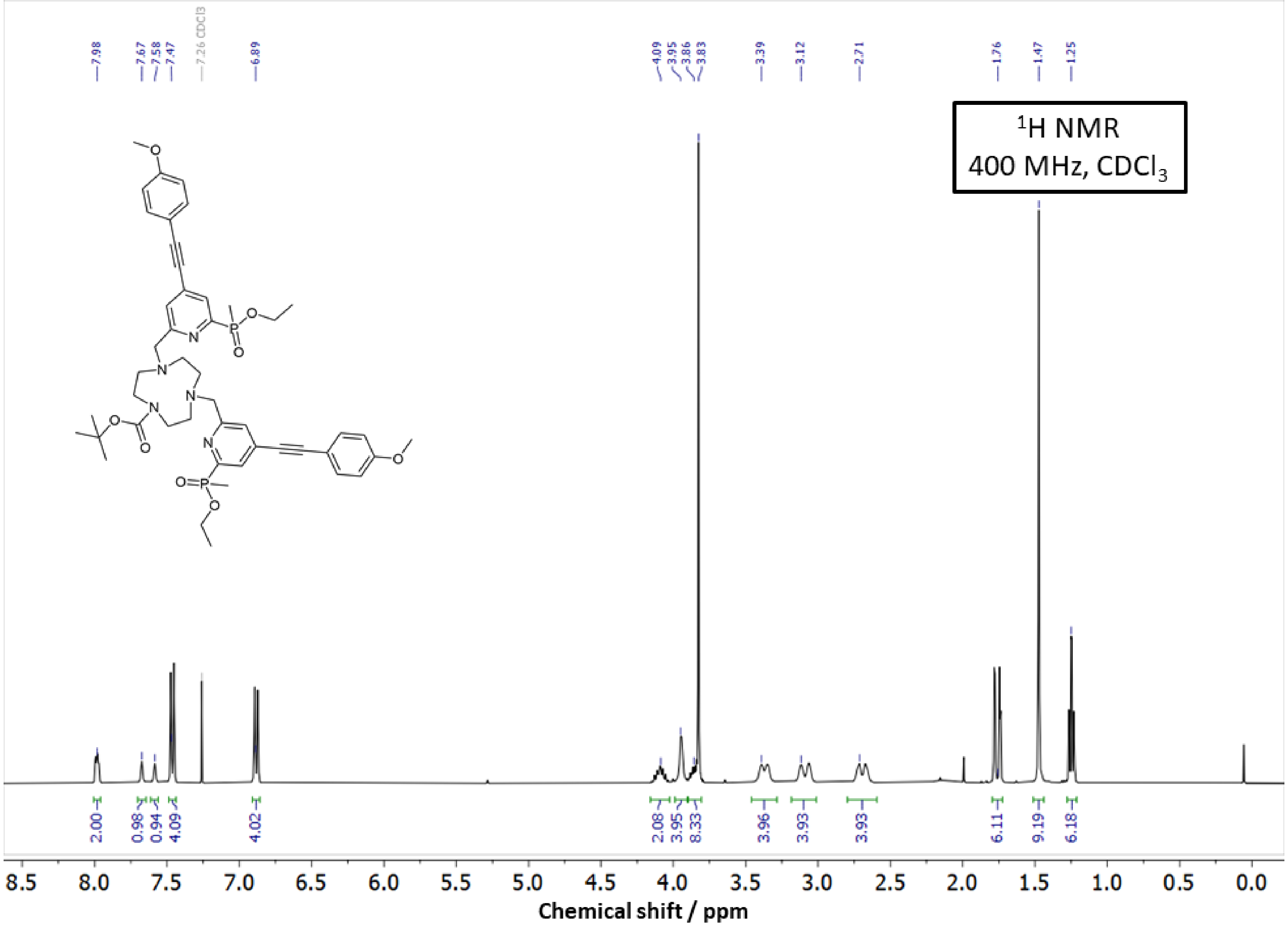

Supplementary Figure 35. ^{1}H NMR (400 MHz, $CDCl_3$) spectrum of compound **9**.

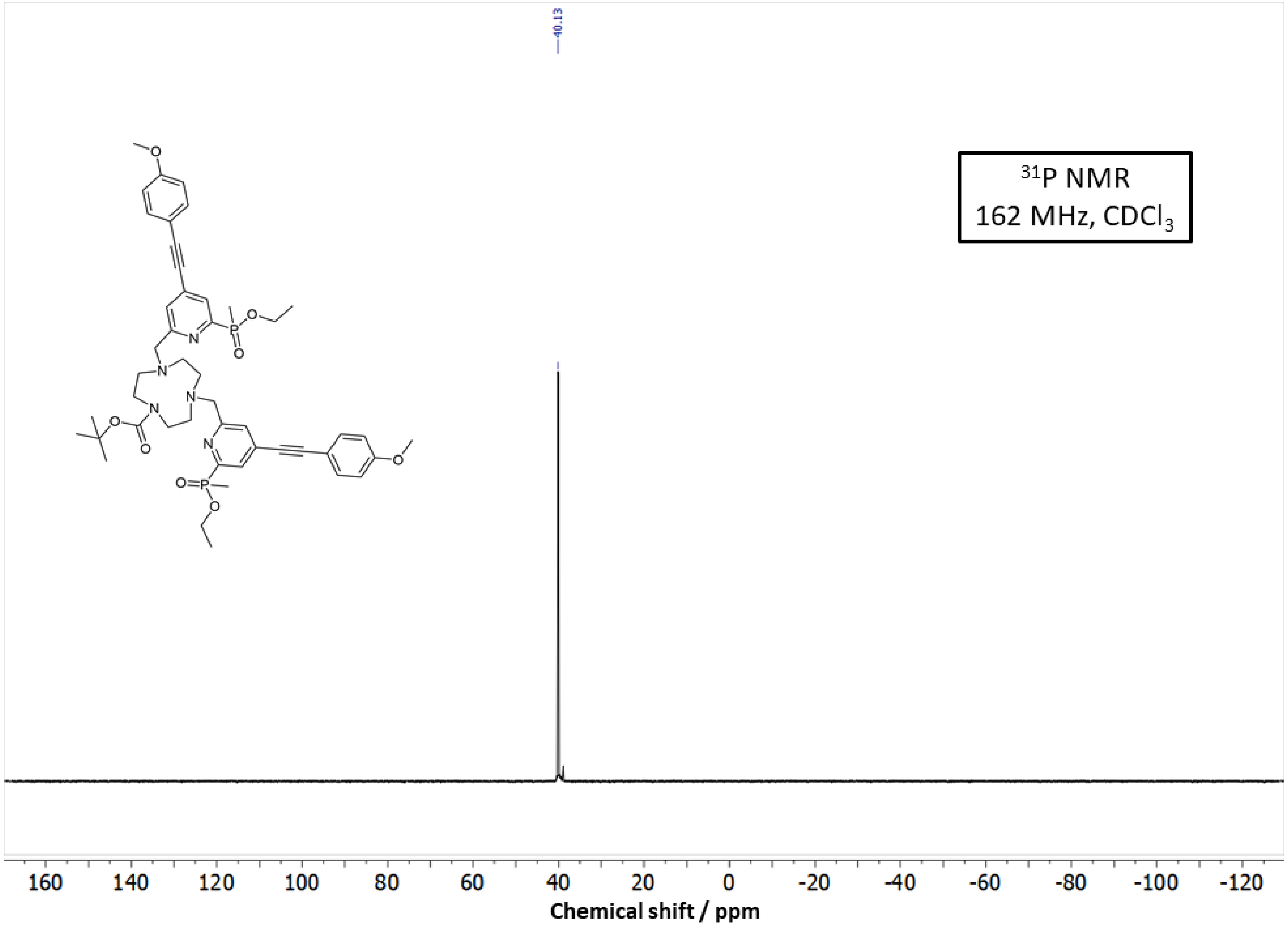

Supplementary Figure 36. ^{31}P NMR (162 MHz, $CDCl_3$) spectrum of compound **9**.

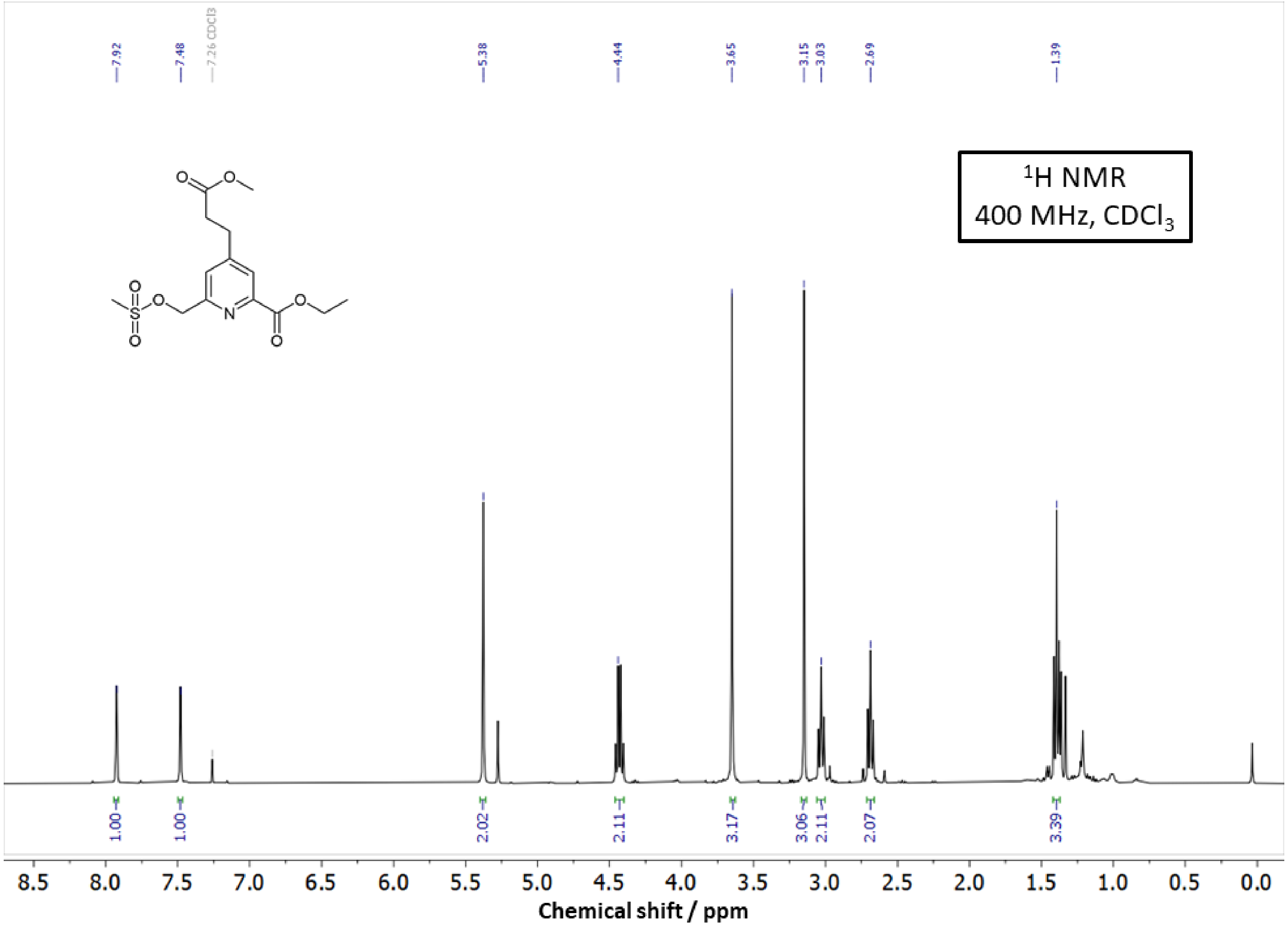

Supplementary Figure 37. ^{1}H NMR (400 MHz, $CDCl_3$) spectrum of compound **11.**

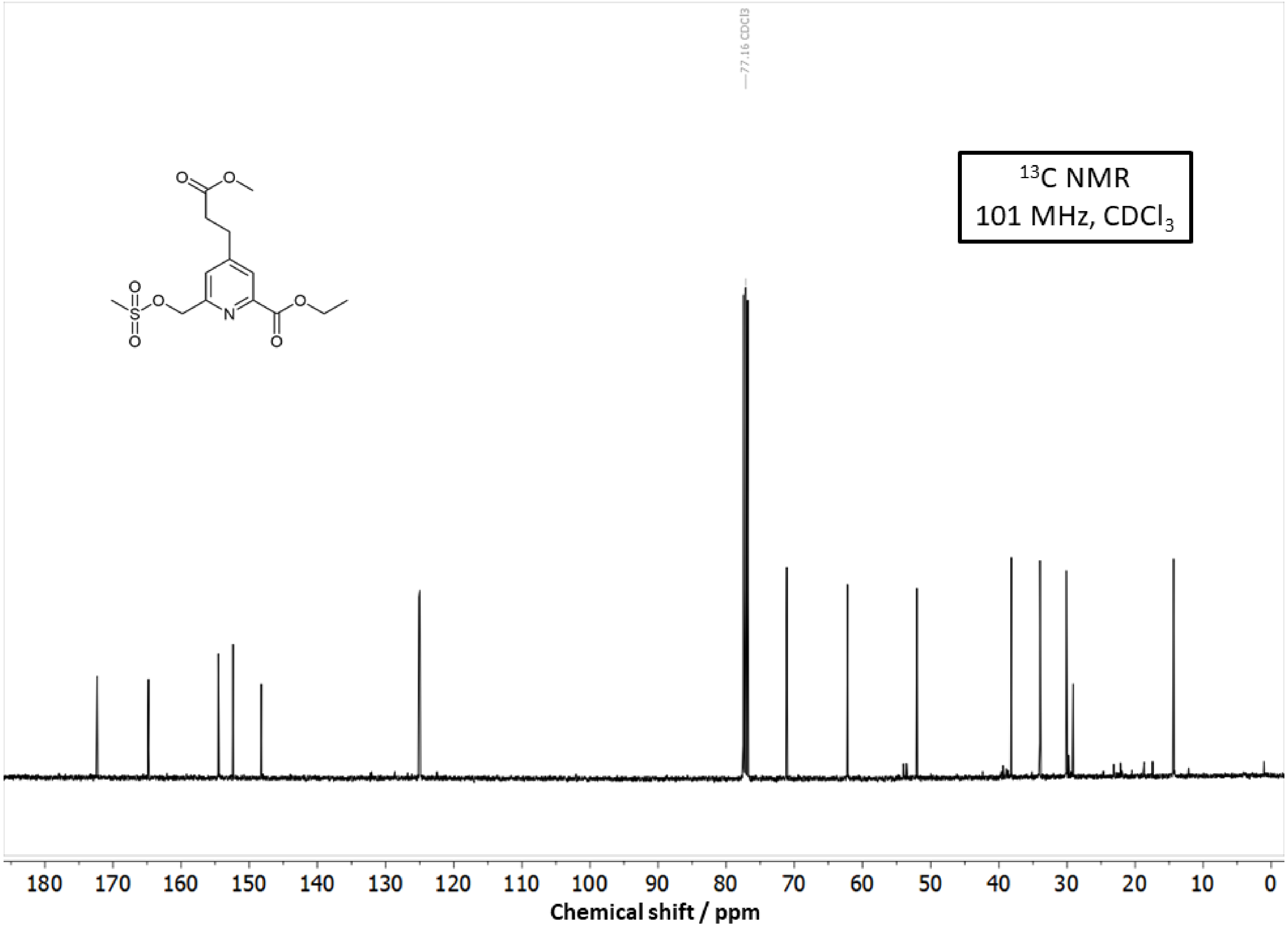

Supplementary Figure 38. ^{13}C NMR (101 MHz, $CDCl_3$) spectrum of compound **11**.

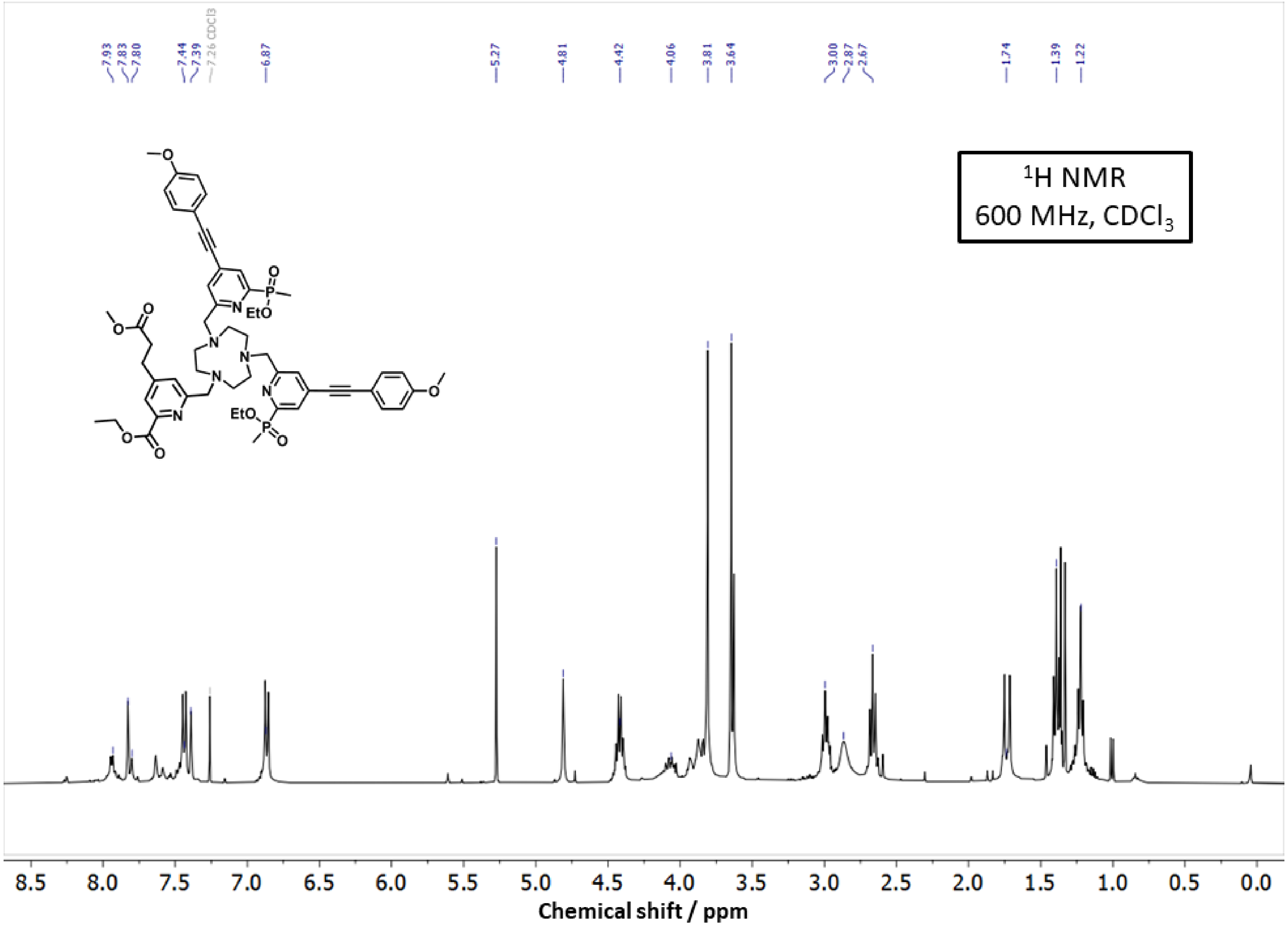

Supplementary Figure 39. Crude ^{1}H NMR (600 MHz, $CDCl_3$) spectrum of ***pro*-L1**.

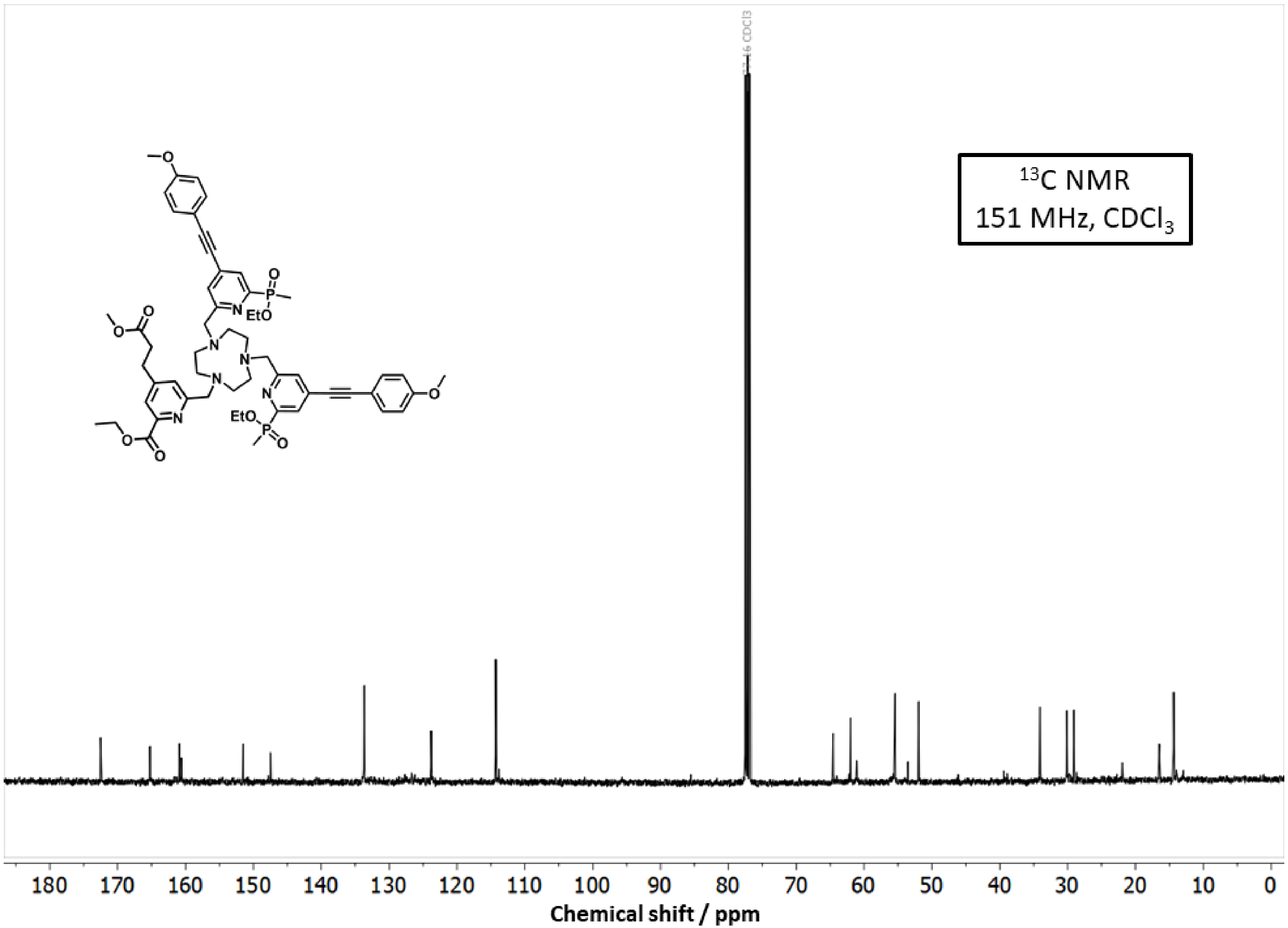

Supplementary Figure 40. Crude ^{13}C NMR (151 MHz, $CDCl_3$) spectrum of ***pro*-L1**.

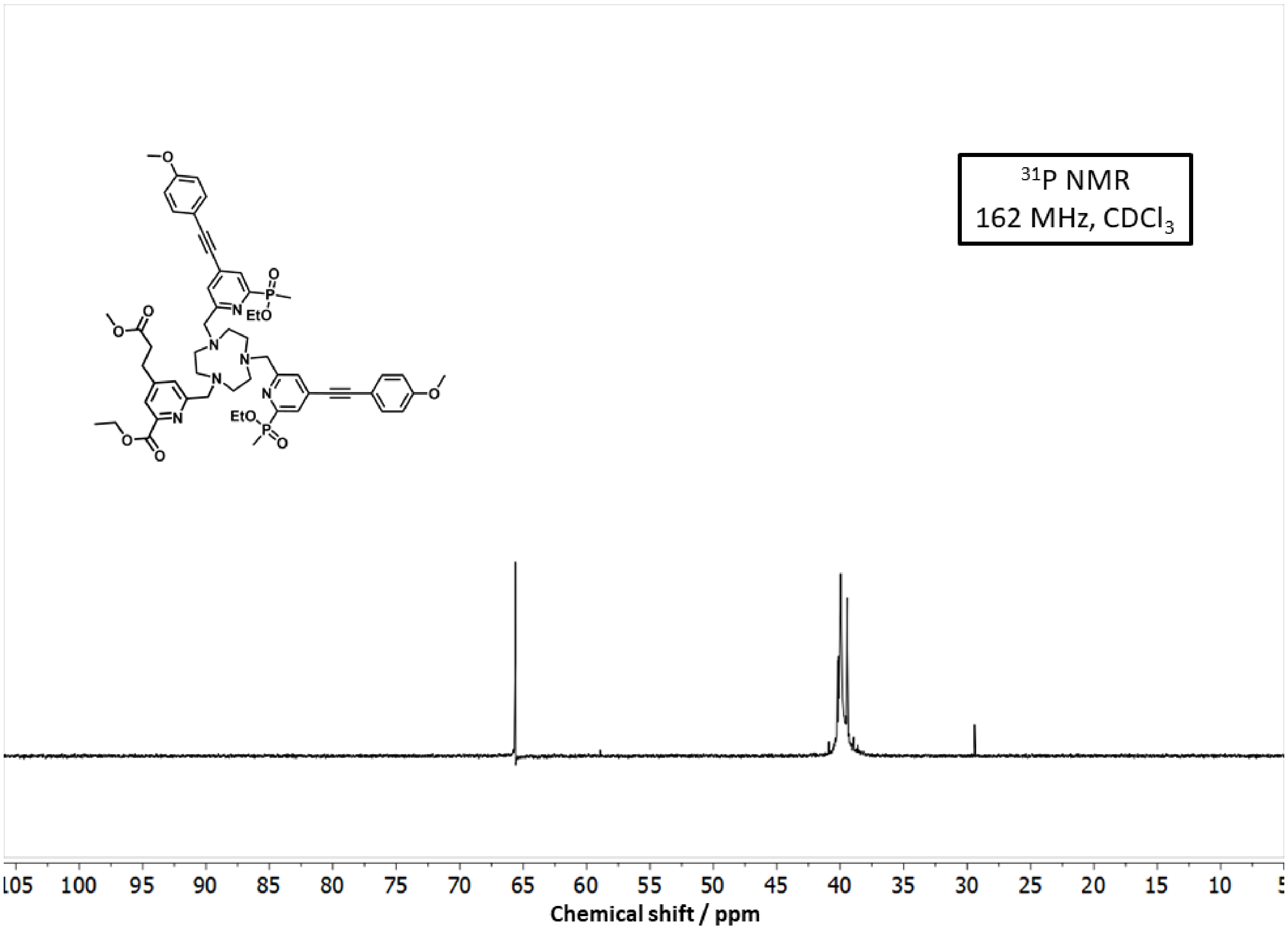

Supplementary Figure 41. Crude ^{31}P NMR (162 MHz, $CDCl_3$) spectrum of ***pro*-L1**

Supplementary References

(1) Frawley, A. T.; Pal, R.; Parker, D. Very Bright, Enantiopure Europium(III) Complexes Allow Time-Gated Chiral Contrast Imaging. *Chem. Commun.* **2016**, *52* (91), 13349–13352.
(2) Montgomery, C. P.; New, E. J.; Parker, D.; Peacock, R. D. Enantioselective Regulation of a Metal Complex in Reversible Binding to Serum Albumin: Dynamic Helicity Inversion Signalled by Circularly Polarised Luminescence. *Chem. Commun.* **2008**, No. 36, 4261.
(3) Carr, R.; Puckrin, R.; McMahon, B. K.; Pal, R.; Parker, D.; Pålsson, L.-O. Induced Circularly Polarized Luminescence Arising from Anion or Protein Binding to Racemic Emissive Lanthanide Complexes. *Methods Appl. Fluoresc.* **2014**, *2* (2), 024007. (*a custom-built instrument based on* Steinberg, I.Z.; Gafni A. Sensitive Instrument for the Study of Circular Polarization of Luminescence. *Rev. Sci. Inst.* **1972**, 43, 409.)
(4) MacKenzie, L. E.; Pålsson, L.-O.; Parker, D.; Beeby, A.; Pal, R. Rapid Time-Resolved Circular Polarization Luminescence (CPL) Emission Spectroscopy. *Nat Commun* **2020**, *11* (1), 1676.
(5) Pal, R.; Beeby, A. Simple and Versatile Modifications Allowing Time Gated Spectral Acquisition, Imaging and Lifetime Profiling on Conventional Wide-Field Microscopes. *Methods Appl. Fluoresc.* **2014**, *2* (3), 037001.
(6) Stachelek, P.; MacKenzie, L.; Parker, D.; Pal, R. Circularly Polarised Luminescence Laser Scanning Confocal Microscopy to Study Live Cell Chiral Molecular Interactions. *Nat Commun* **2022**, *13* (1), 553.
(7) Starck, M.; MacKenzie, L. E.; Batsanov, A. S.; Parker, D.; Pal, R. Excitation Modulation of Eu:BPEPC Based Complexes as Low-Energy Reference Standards for Circularly Polarised Luminescence (CPL). *Chem. Commun.* **2019**, *55* (94), 14115–14118.
(8) Xu, C.; Webb, W. W. Measurement of Two-Photon Excitation Cross Sections of Molecular Fluorophores with Data from 690 to 1050 Nm. *J. Opt. Soc. Am. B* **1996**, *13* (3), 481.
(9) Pålsson, L.-O.; Pal, R.; Murray, B. S.; Parker, D.; Beeby, A. Two-Photon Absorption and Photoluminescence of Europium Based Emissive Probes for Bioactive Systems. *Dalton Trans.* **2007**, No. 48, 5726.
(10) Pal, R. Phase Modulation Nanoscopy: A Simple Approach to Enhanced Optical Resolution. *Faraday Discuss.* **2015**, *177* (0), 507–515.
(11) Takalo, H.; Kankare, J.; Lund, H.; Makmur, L.; Norrestam, R. Synthesis of Dimethyl and Diethyl 4-(Phenylethynyl)-2,6-Pyridinedicarboxylate. *Acta Chemica Scandinavica - ACTA CHEM SCAND* **1987**, *41*, 219–221.
(12) Lamarque, L.; Parkjer D.; Butler, S.J.; Delbianco, M. US Pat., US 20150361116 A1: Novel water-soluble complexing agents and corresponding lanthanide complexes. 2016.
(13) Starck, M.; Fradgley, J. D.; Vita, S. D.; Mosely, J. A.; Pal, R.; Parker, D. Targeted Luminescent Europium Peptide Conjugates: Comparative Analysis Using Maleimide and Para-Nitropyridyl Linkages for Organelle Staining. *Bioconjugate Chemistry* **2019**.
(14) Kovacs, Z.; Sherry, A. D. A General Synthesis of Mono- and Disubstituted 1,4,7-Triazacyclononanes. *Tetrahedron Letters* **1995**, *36* (51), 9269–9272.
